# Incidence of Dengue Fever in Pakistan

**DOI:** 10.1371/journal.pone.0352938

**Published:** 2026-07-02

**Authors:** Zarmeen Nasim, Robert C. Reiner, Sana Sheikh, Nida Saddaf Khan, Mushyada Ali, Isbaah Tejani, Mirza Tayyab Mehmood, Javerya Hassan, Khalid Aziz, Joveria Farooqi, Najia Ghanchi, Erum Khan, Rafey Ali, Imran Ahmed Chauhadry, Aun Raza, Sadia Hassan, Faisal Sultan, Omar Chughtai, Hijab Batool, Ali Rehman, Mumtaz Ali Khan, Samuel Ostroff, Ali H. Mokdad, Nosheen Nasir, Zainab Samad, Bilal Ahmed Usmani

**Affiliations:** 1 CITRIC Health Data Science Centre, The Aga Khan University, Karachi, Pakistan; 2 Institute for Health Metrics and Evaluation, Seattle, Washington, United states of America; 3 Department of Medicine, The Aga Khan University, Karachi, Pakistan; 4 McWilliams School of Biomedical Informatics, University of Texas Health Science Center, Houston, Texas, United States of America; 5 Department of Pathology & Laboratory Medicine, The Aga Khan University, Karachi, Pakistan; 6 Department of Paediatrics and Child Health, The Aga Khan University, Karachi, Pakistan; 7 Shaukat Khanum Memorial Cancer Hospital and Research Centre, Lahore, Pakistan; 8 Chemical Pathology, Chughtai Institute of Pathology, Lahore, Pakistan; 9 Integrated Disease Surveillance and Response System, National Institute of Health, Islamabad, Pakistan; 10 Centre for Disease Control (CDC), National Institutes of Health, Islamabad, Pakistan; 11 Department of Community Health Sciences, The Aga Khan University, Karachi, Pakistan; King Faisal University, SAUDI ARABIA

## Abstract

**Introduction:**

Dengue is a growing health threat in Pakistan due to climate, urbanization, and inadequate sanitation. Despite recurrent seasonal outbreaks, comprehensive evidence on the dengue burden in Pakistan remains limited due to due to underreporting, incomplete data capture, and inconsistencies in diagnostic practices.

**Methods:**

We analyzed laboratory-confirmed dengue cases from January 2012 to December 2022 to estimate incidence rates per 1,000 individuals across Pakistan at the subnational level including all 4 provinces, 86 districts, and a federal territory, stratified by 5 distinct age groups (below 5 years, 5–14 years, 15–49 years, 50–69 years, and 70 + years) and sex. The population estimates were adjusted to account for variations in treatment-seeking behavior and the utilization of partnering laboratories to ensure that the estimated incidence rates accurately represent the broader population.

**Results:**

The incidence rate of dengue in Pakistan exhibited an increasing trend. Across all age groups, the 15–49 years age group had the highest incidence rates, with 56 cases per 1,000 individuals in 2022 (estimated 1.6 million cases). Sex analysis showed a higher proportion of dengue cases in males compared to females, consistent across all age groups. Spatial analysis revealed regional disparities, with higher incidence rates reported from Sindh compared to other provinces. In 2022, Sindh had the highest incidence rate of 45.63 (95% UI: 34.13–60.68) per 1000 individuals (2.63 million estimated cases), while Baluchistan had the lowest incidence rates of 0.44 (95% UI: 0.08–4.48) per 1,000 individuals (6,785 estimated cases). All age groups experienced increasing incidence, with the 15–49 age group showing the most pronounced rise.

**Conclusion:**

The findings underscore the growing burden of dengue in Pakistan, highlighting the urgent need for effective public health interventions and enhanced data collection mechanisms to better understand and address this rising public health concern.

## Introduction

Dengue fever, a mosquito-borne viral infection caused by the dengue virus (DENV), poses a significant global health threat, particularly in tropical and subtropical regions. It can present with a range of symptoms that include high fever, headaches, skin rashes, nausea, enlarged lymph nodes, eye pain, and severe muscle and joint pain [1]. According to the World Health Organization (WHO) classification, dengue is categorized into dengue without warning signs, dengue with warning signs, and severe dengue [2]. Severe dengue can lead to complications such as organ failure and hemorrhage, posing a significant risk of mortality if not treated in a timely manner [3]. A secondary infection is also associated with an increased risk of progression to severe disease, likely due to immunological mechanisms such as antibody-dependent enhancement [4]. There are an estimated 390 million infections annually worldwide with many studies projecting a sustained increase in the number of cases over time, partly attributed to climate change [5,6]. This trend persists despite efforts to combat and mitigate its impact including WHO’s global vector control response which aims to reduce mortality attributed to dengue fever by 75% [7]. Recently, dengue vaccines such as CYD-TDV (Dengvaxia) and TAK-003 (Qdenga) have been approved in several countries, although their use is subject to specific recommendations based on serostatus and age [8].

Pakistan, a country in South Asia of over 230 million people, is grappling with the challenges posed by dengue. The fifth-most populous country in the world, Pakistan is home to densely populated urban centers such as Karachi, Lahore, and Faisalabad, which are conducive environments for the proliferation of the *Aedes aegypti* mosquito vectors responsible for the majority of dengue virus transmission [9]. Factors such as climate change, the rise in urban populations, and inadequate sanitation contribute to the rising incidence of dengue fever [10]. The World Health Organization (WHO) reported the first case of dengue fever (DF) in Pakistan in 1994, representing the earliest confirmed report of the disease in the country, although earlier circulation may have gone undetected due to limited surveillance and diagnostic capacity [11–13]. Pakistan experiences annual outbreaks of dengue; cases increase during the post-monsoon season from September to November. In 2010−11, Lahore had a severe dengue outbreak with over 21,000 reported cases and 352 deaths [14]. Notably, in 2021, there was a significant surge in cases observed in Punjab specifically in Lahore, Rawalpindi, and Islamabad, with confirmed cases reaching 48,906 [15]. In 2022, Pakistan had devastating floods that resulted in the outbreak of dengue and other vector-borne diseases [16] with 25,932 confirmed cases. Despite these significant reports, comprehensive and consistent epidemiological data on the overall burden of dengue in Pakistan remains limited. Another notable challenge in estimating disease incidence arises from the fact that majority of dengue infections are asymptomatic causing many individuals to avoid seeking medical care [17]. This results in asymptomatic and clinically inapparent infections that contribute to ongoing transmission while remaining undetected in routine surveillance. Existing surveillance data often suffer from underreporting, incomplete data capture, and inconsistencies in diagnostic practices, hindering accurate estimation of disease incidence and associated morbidity and mortality [18]. Furthermore, the spatial and temporal variability of dengue outbreaks across different regions of Pakistan necessitates tailored approaches for disease monitoring, prevention, and control [9].

In this study, we addressed these knowledge gaps by estimating the incidence and incidence rates of dengue fever in Pakistan by age, sex, and location between 2012 and 2022. We leveraged data from three nation-wide high-volume private lab networks that operate across Pakistan. These laboratories were selected due to their extensive reach and high-quality diagnostic capabilities. The data, accessed through collaboration with these lab networks, was used to estimate the trends, spatial distribution, and incidence rate of dengue fever in Pakistan across four (4) provinces, one (1) federal territory, and eighty-six (86) districts. Through statistical modeling and spatial analysis techniques, we aimed to generate reliable estimates of dengue incidence rates by age, sex, and location and identified high-risk areas that require greater resource mobilization and coordinated dengue prevention control mechanisms than currently available.

## Methods

### Ethics statement

The study was approved by the Ethical Review Committee (ERC) of Aga Khan University (Ref# 2023-9439-27198) and an exemption was obtained. Separate institutional review board approvals were obtained from all participating laboratories prior to data curation, including the Institutional Review Board of Chughtai Institute of Pathology, Lahore (CIP/IRB/1358), and the Institutional Review Board of Shaukat Khanum Memorial Cancer Hospital and Research Centre, Lahore (EX‑05‑11‑23‑01). All data accessed for this study were fully anonymized.

### Study design and setting

This retrospective research estimated the incidence of dengue fever in Pakistan using laboratory-based data collected from three large lab networks (Lab A: 300 + units, Lab B: 250 + units, Lab C: 440 units) functioning across the country. A collection unit refers to the laboratory facility where the sample was collected. The deidentified data, covering the period from January 2012 to December 2022, was requested from the lab networks in April 2024. The raw data were available at the patient level and were subsequently aggregated at the monthly level to assess temporal trends, and at the annual level to generate incidence estimates. The data from Lab A and B was used to generate incidence estimates, while Lab C data was used for trend validation. The study adheres to the STROBE guidelines, and the checklist is provided in supplementary appendix (S1 Table).

### Data collection

We collected deidentified data from the three lab networks’ electronic medical records (EMRs). A standardized data template provided in supplementary appendix (S2 Table) was shared with the partnering labs to ensure data consistency across three sites. Data included patient demographics, test type (NS1 antigen and IgM), results and visit date. A case was considered positive for dengue if either the NS1 antigen or IgM test was positive.

### Data preprocessing

**Fig 1:**
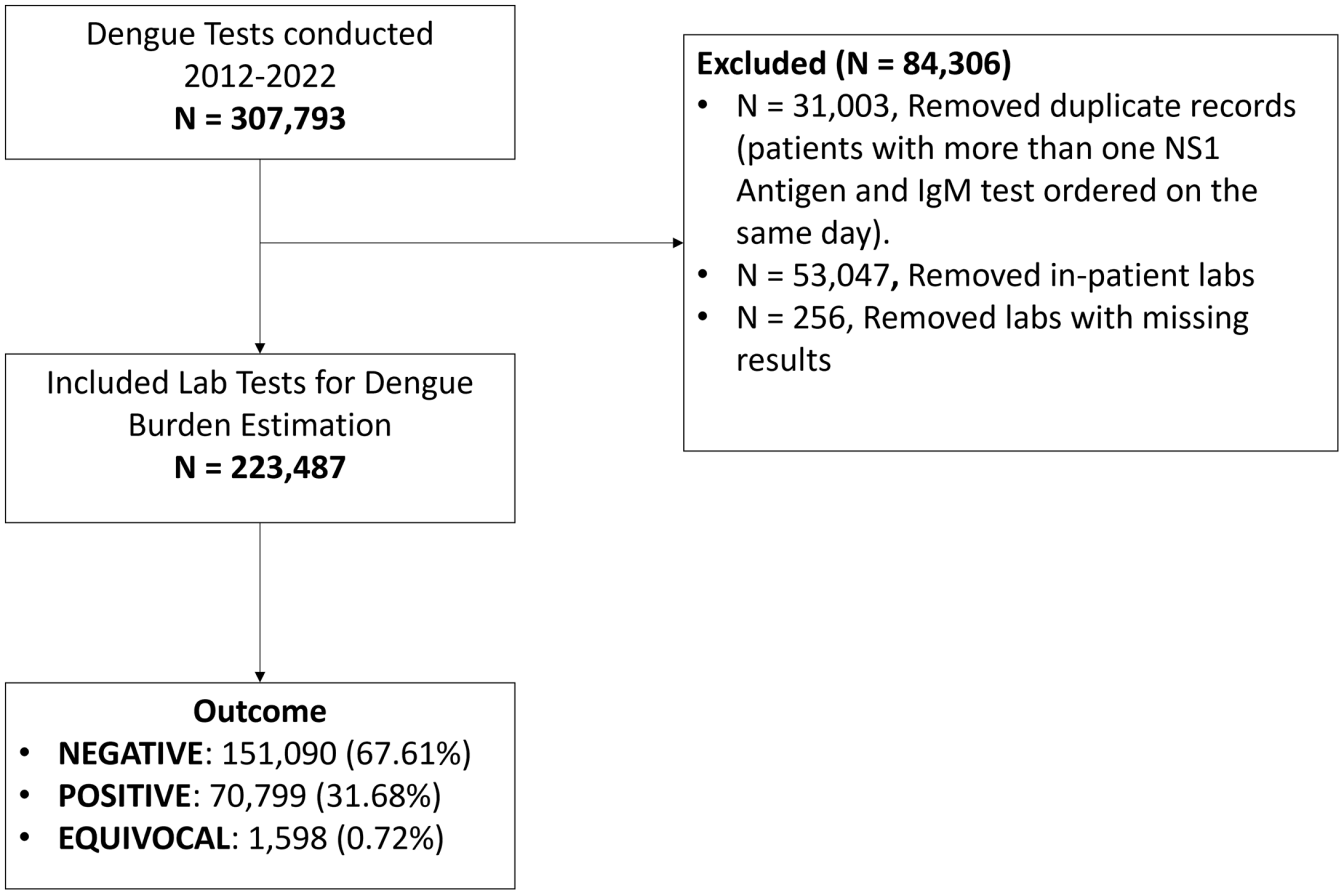
Workflow of Study.

During the data preprocessing, several key operations were performed to ensure data quality and consistency. To address data redundancy, if multiple dengue tests were conducted during the same visit (e.g., NS1 and IgM), the result was recorded as positive if either test was positive; otherwise, it was marked as negative. Fig 1 presents the workflow of study. Data analysts in the team extracted and manually verified geo coordinates of the collection point using Google Map API [19].

### Statistical methods

We analyzed the trend of dengue disease during the study period and identified peaks indicating the outbreaks. Temporal trends were assessed using the Seasonal Mann – Kendall test applied to monthly data to account for dengue seasonality, with trend magnitude estimated using Sen’s slope. Trend analyses were conducted at both the national and provincial levels. We then examined the age and sex distribution of laboratory-confirmed dengue positive cases over the study period. The differences in case distributions across age groups and between sexes were assessed using Chi-Square Test of Independence. Age-specific cumulative incidence rates were calculated per 1000 population, and the differences across age groups (below 5, 5–14, 15–49, 50–69, and 70+) were further evaluated using Poisson regression. We also calculated estimates for incidence rate of dengue across subnational locations of Pakistan, including 4 provinces, 86 districts, and a federal territory. We estimated the annual incidence rate of dengue at the district level per 1000 persons using the laboratory-confirmed cases as the numerator (See Equation 1).


$$\begin{array}{c} {Incidence\;of\;Dengue\;Per\;\text{1}\text{0}\text{0}\text{0}}_{District,Year}=\;\frac{{New\;Dengue\;Cases\;}_{District}}{\;Adjusted\;{Mid\;year\;population}_{District,Year}}\;*\;\text{1}\text{0}\text{0}\text{0} \end{array}$$ (1)

The numerator is derived from the number of new dengue-positive cases identified at the two partnering labs (Lab A and B) within each district, based on diagnostic tests (NS1 antigen/IgM). The denominator that represents the total number of individuals who may have sought dengue testing was determined after applying a few adjustments (Equation 2) to the catchment population of a district.

### Estimating the catchment population in a district

For the denominator, we focused on the distribution of collection units (laboratories) across the districts. Districts with five or more collection units were considered to have densely distributed facilities. To justify the ≥ 5 threshold, we conducted a spatial accessibility analysis using GPS coordinates of all collection units. For each 100 m² population pixel, we calculated distances to the nearest collection unit and overlaid these with population data to compute population-weighted mean distances. Our analysis (Supplementary S1 Fig) showed that districts with ≥5 collection units had substantially higher population coverage within short travel distances (e.g., 5–10 km), with coverage approaching saturation in districts with ≥10 collection units. In contrast, districts with 1–4 collection units exhibited lower and more variable coverage. Based on these findings, districts with ≥5 collection units were assigned the full mid-year district population from the national census as the catchment population. For districts with fewer than five units, we estimated the catchment population within a 5 km radius of each collection unit using ArcGIS software [20].

### Adjusting the catchment population

To generate robust estimates of incidence rates, we applied several adjustments to the denominator to account for variations in healthcare-seeking behavior and laboratory testing practices. These adjustments included: (i) treatment-seeking rate for fever, (ii) likelihood of obtaining a blood test following fever, and (iii) estimated proportion of the population using the partnering labs for testing. According to Pakistan Demographic and Health Survey [21], 81.4% people seek physician care for fever in Pakistan. We applied provincial estimates available for treatment seeking for children (<5 years) in case of fever [21]. In addition, we estimated that 15.3% (95% CI: 12.13% − 18.47%) of individuals with fever obtain blood testing [22]. As direct data on private healthcare utilization was unavailable, we relied on estimates from comparable settings, ranging from 6% to 10%. We adopted a conservative estimate of 6% for the population utilizing partnering lab facilities [23]. All of these adjustments were applied to estimate the subset of district population likely to undergo dengue testing at the partnering labs (Equation 2).


$$\begin{array}{c} Adjusted\;{Mid\;year\;population}_{District,Year}=P\;\times \;T_{prov}\;\times \;R_{test}\times L_{util} \end{array}$$ (2)

where,

- P is the total population of the district in a year.
- $T_{prov}\;$is the treatment-seeking rate for fever (as a proportion), based on the Pakistan Demographic and Health Survey values:
  - Punjab: 0.853
  - Sindh: 0.847
  - Khyber Pakhtunkhwa: 0.715
  - Baluchistan: 0.594
  - Islamabad: 0.738
- $R_{test}$ is the proportion of individuals who obtain blood testing following fever: 0.153 (95% CI: 0.121–0.185).
- $L_{util}$ is the estimated proportion of the population that utilizes the partnering lab facilities for testing: 0.06

We conducted a sensitivity analysis for Sindh province in year 2022, varying $T_{prov}$, $R_{test}$ (within its 95% CI), and $L_{util}$ (6%−10%). Incidence rate of dengue per 1000 persons and the corresponding 95% uncertainty intervals were recalculated for each scenario.

Similarly, to estimate the age specific incidence rate (ASI) of dengue at provincial and district levels, we considered dengue positive cases in a specific age group as the numerator and the adjusted age-specific population as the denominator (See Equation 3). The same adjustments were applied as in the overall incidence rate estimation. District populations were estimated through Inter-Census estimation method [24], utilizing publicly available censuses from 1998, 2017 and 2023.


$$\begin{array}{c} {ASI\;of\;Dengue\;Per\;\text{1}\text{0}\text{0}\text{0}}_{District,Year}=\;\frac{{New\;Dengue\;Cases\;}_{Age\;specific,\;District}}{\;{Adjusted\;Mid\;year\;population}_{Age\;specific,\;District,Year}}\;*\;\text{1}\text{0}\text{0}\text{0} \end{array}$$ (3)

## Results

### Overview of collection units and dengue testing

Collection units of the partnering labs were present across the country as shown in S2 Fig. Most of these units were present in Sindh and Punjab provinces. Gilgit Baltistan, Azad Kashmir and Balochistan have fewer lab units consistent with the size of the population in these areas. The trends in dengue testing across Pakistan from 2012 to 2022 showed regional disparities, with noticeable increases in testing primarily in Sindh and Punjab. In contrast, testing remains limited in Baluchistan, KPK, Islamabad, Gilgit-Baltistan, and Azad Kashmir. S3 Fig illustrates the non-uniformity in the testing.

### Age and sex distribution of dengue positive cases (2012–2022)

The population distribution of Pakistan across the five age groups were estimated from Population Pyramid [25]. Of the study population, 13.05% were younger than 5 years, 24.4% were aged 5–14 years, 49.9% were aged 15–49 years, 10.25% were aged 50–69 years, and 2.4% were elderly individuals aged 70 years and above.

S4 Fig illustrates the distribution and cumulative incidence rates of dengue cases across five age groups from 2012 to 2022. The highest number of cases were found in the 15–49 years age group, with 45,390 cases (64.11% of total cases) and a cumulative incidence rate of 60.20 per 1000. The 50–69 years group had 9,246 cases (13.06%) with a similar incidence rate of 59.70 per 1000 (p = 0.464). Children ≤5 years had 3,356 cases (4.74%) with an incidence rate of 17.02 per 1000, the 5–14 years group had 11,073 cases (15.64%) with 30.03 per 1000, and adults ≥70 years had 1,734 cases (2.45%) with 47.82 per 1000; all three age groups had significantly lower cumulative incidence rates in contrast to the 15–49 years group (p < 0.001).

The sex distribution of laboratory-confirmed dengue cases differed significantly across age groups (χ^2^ = 192.49, p < 0.001). As shown in S5 Fig, male constituted a higher proportion of dengue cases in contrast to females, and this pattern was consistent across varying age groups.

### Trend analysis

**Fig 2:**
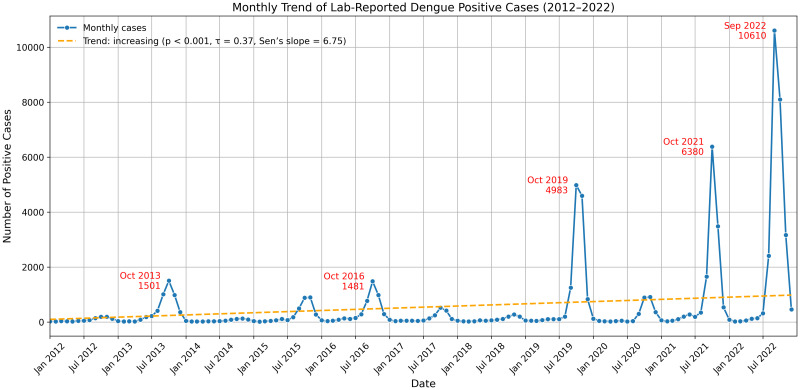
Trend Analysis of Laboratory Confirmed Dengue Positive Cases during the Study Period (January 2012-December 2022).

The trend analysis indicated seasonal variations in the number of laboratory-confirmed dengue positive cases over the study period (January 2012 – December 2022), with recurrent peaks during post-monsoon seasons (September-October-November). Notable surges were observed in 2019, 2021, and 2022. Specifically, the peak monthly case count increased by 28% from October 2019 (4,983 cases) to October 2021 (6,380 cases), and by 66.3% from October 2021 to September 2022 (10,610 cases). In the Seasonal Mann–Kendall test, we found a statistically significant increasing trend in monthly dengue cases over time (p < 0.001, tau = 0.37), after accounting for seasonal effects. The estimated Sen’s slope indicated an average increase of approximately 6.75 dengue-positive cases per month. Province-level trends were heterogeneous, with significant increases in Sindh (p < 0.001) and Khyber Pakhtunkhwa (p = 0.002), while other provinces showed no significant trends (S6 Fig). Fig 2 shows the total number of dengue confirmed cases recorded monthly during the study period.

We also analyzed the baseline trend of dengue cases to understand the endemicity, excluding peak season (September–October–November) during the outbreak years. Outbreak years, including 2019 and 2022, were identified using a residual-based approach, where months with observed case counts exceeding Sen’s slope-based expectations by more than two standard deviations for at least two consecutive months were classified as outbreak periods, consistent with prior literature [26]. S7 Fig (See supplementary appendix) shows the average number of dengue positive cases in a season across the study period. Our analysis indicated a consistent presence of the disease even during non-peak seasons.

### Incidence of dengue in Pakistan

The analysis showed an increase in incidence of dengue cases from an estimated 0.1 million cases in Year 2012 to 3.28 million cases in Year 2022 across Pakistan. The estimates showed that Sindh with 2.63 million cases (45.63 [95% UI: 34.13–60.68] per 1000) had the highest incidence of the disease in Year 2022 in contrast to other provinces and territories in Pakistan.

**Fig 3:**
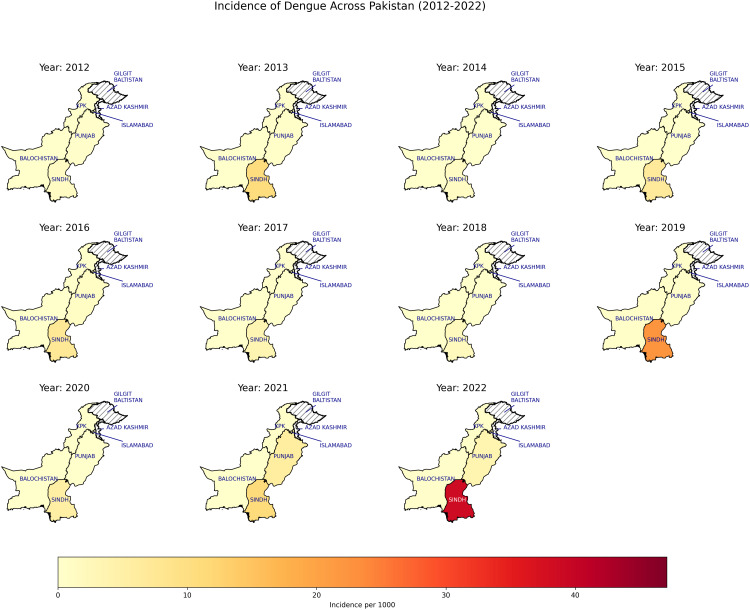
Incidence of Dengue in Pakistan from 2012 to 2022 Across Provinces and Federal Territories. Shape file source: World Food Programme SDI, URL: https://data.humdata.org/dataset/cod-em-pak under a CC BY license.

Dengue incidence rates varied considerably across the country, indicating geographic and temporal disparities. Fig 3 illustrates the annual incidence rates, with Sindh consistently highest, peaking in 2019 (26.36 [95% UI: 18.10–38.34] per 1000 cases extrapolated to 1.49 million cases) and 2022 (45.63 [95% UI: 34.13–60.68] per 1000 cases extrapolated to 2.63 million cases). Punjab shows moderate to high incidence rates with significant increases, particularly in 2016 (1.43 [95% UI: 0.37–6.44] per 1000 cases extrapolated to 0.15 million cases) and 2021 (7.09 [95% UI: 3.67–14.86] per 1000 extrapolated to 0.93 million cases). Balochistan exhibited lower incidence rates throughout the study period. We observed the increasing trend over the 11-year period, with the steepest increases occurring in the last few years. Table 1 provides incidence rate estimates for all subnational locations.

**Table 1:** Incidence Rate Per 1000 of Dengue across Selected Subnational Regions in Pakistan.

Years	Incidence Rate per 1000				
	SINDH	PUNJAB	BALOCHISTAN	KPK	ISLAMABAD
2012	1.71[95% UI: 0.45–6.71]	0.40 [95% UI: 0.08 - 4.43]	0.05[95% UI: 0.03–4.04]	0.09[95% UI: 0.04–3.74]	0.09[95% UI: 0.04–3.66]
2013	12.52[95% UI: 7.01–21.52]	0.49[95% UI: 0.1–5.12]	0.15[95% UI: 0.04–3.85]	0.45[95% UI: 0.08–4.69]	3.46[95% UI: 1.45–9.68]
2014	1.60[95% UI: 0.46–6.52]	0.08[95% UI: 0.03–3.74]	0.03[95% UI: 0.02–3.71]	0.08[95% UI: 0.03–3.88]	0.08[95% UI: 0.04–4.04]
2015	7.92[95% UI: 4.05–15.54]	0.20[95% UI: 0.05–3.64]	0.08[95% UI: 0.03–3.78]	0.28[95% UI: 0.06–4.08]	0.24[95% UI: 0.04–4.27]
2016	8.61[95% UI: 4.53–15.85]	1.44[95% UI: 0.37–6.45]	0.17[95% UI: 0.04–3.82]	0.19[95% UI: 0.04–3.69]	0.38[95% UI: 0.08–4.24]
2017	4.01[95% UI: 1.61–10.3]	0.19[95% UI: 0.06–3.9]	0.15[95% UI: 0.05–4.35]	0.45[95% UI: 0.09–4.71]	0.15[95% UI: 0.05–4.08]
2018	2.41[95% UI: 0.87–7.81]	0.07[95% UI: 0.04–3.86]	0.15[95% UI: 0.05–3.93]	0.04[95% UI: 0.04–3.92]	0.12[95% UI: 0.05–3.84]
2019	26.37[95% UI: 18.1–38.34]	0.46[95% UI: 0.09–4.86]	0.68[95% UI: 0.14–4.77]	0.74[95% UI: 0.19–5.33]	4.49[95% UI: 1.78–10.14]
2020	6.17[95% UI: 2.74–13.02]	0.05[95% UI: 0.03–3.85]	0.08[95% UI: 0.02–3.37]	0.04[95% UI: 0.03–3.91]	0.00[95% UI: 0.03–3.68]
2021	12.95[95% UI: 7.88–22.34]	7.1[95% UI: 3.68–14.86]	0.15[95% UI: 0.05–4.4]	0.70[95% UI: 0.19–4.88]	0.79[95% UI: 0.18–5.45]
2022	45.63[95% UI: 34.14–60.68]	4.47[95% UI: 1.9–10.49]	0.44[95% UI: 0.08–4.48]	0.85[95% UI: 0.19–5.24]	5.52[95% UI: 2.54–12.67]

The robustness of dengue incidence estimates of Sindh in 2022 were assessed through the sensitivity analysis by varying denominator assumptions, including treatment-seeking rate, blood testing following fever rate, and the proportion utilizing partnering lab facilities (See S8 Fig in Supplementary Appendix).

### Age-specific incidence rate of Dengue in Pakistan

Further, we estimated age-specific incidence per 1000 across subnational regions in Pakistan. S9 Fig (Supplementary Appendix) presents the age-group specific incidence of dengue for the years a) 2012 and b) 2022 across the geographical provinces and territories in Pakistan. Dengue incidence increased across all age groups from 2012 to 2022. In 2012, the incidence was particularly highest in Sindh among the 15–49 (2.49 [95% UI: 0.74–7.99] per 1000 extrapolated to 53,888 cases in the population) and 50–69 (2.09 [95% UI: 0.61–7.18] per 1000 extrapolated to 9,260 cases) age groups. By 2022, dengue cases increased across all age groups, with the 15–49 age group exhibiting the highest incidence of 56 [95% UI: 43.63–70.75] per 1000 cases (1.60 million cases).

### District level estimates of Dengue in 2022

**Fig 4:**
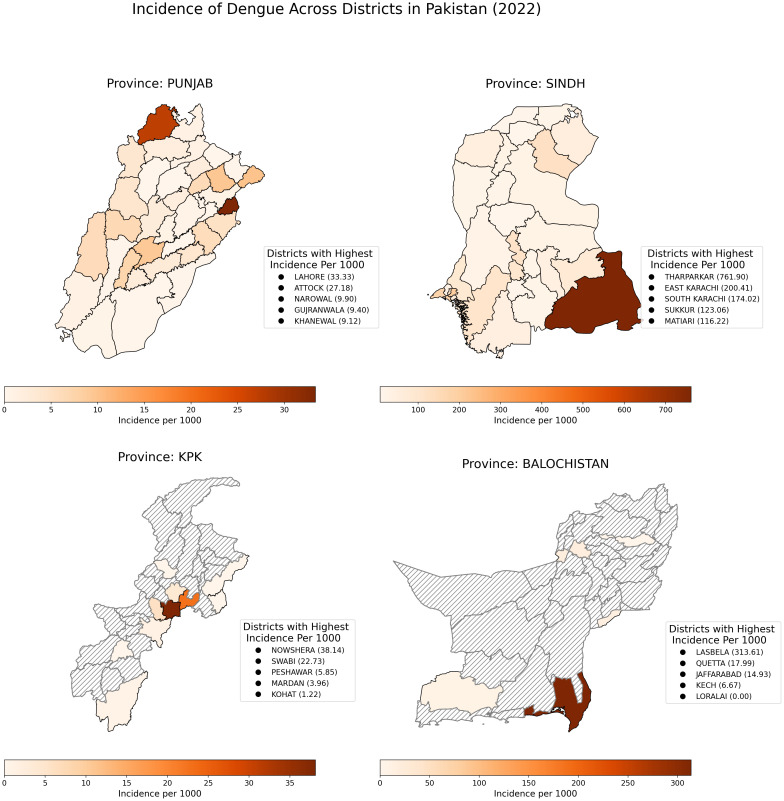
Incidence of Dengue Across Districts. Shape file source: World Food Programme SDI, URL: https://data.humdata.org/dataset/cod-em-pak under a CC BY license.

In addition to age-specific incidence estimates for dengue, we also generated estimates of dengue incidence at a district-level for four subnational locations. Sindh experienced the highest incidence of dengue during the study period. Fig 4 illustrates the distribution of dengue incidence in certain districts across four provinces in the year 2022. The highest incidence of dengue was found in Tharparkar (761.90 [95% UI: 734.6–786.3] cases per 1000 individuals) district of Sindh followed by East Karachi (200.41 [95% UI: 176.40–224.86] per 1000) district located in Sindh. District-level dengue incidence rates per 1,000 over the 11-year period are presented in S3 Table (see appendix).

## Discussion

This study quantitatively assessed the incidence rate of dengue in Pakistan from January 2012 to December 2022, utilizing data from three extensive national laboratory networks. Unlike previous studies, this analysis integrated large-scale laboratory-confirmed data with population-based adjustments to estimate incidence rates at various levels of granularity, including provincial, district, sex, and age group levels. These adjustments were applied to account for known sources of under‑ascertainment. While such adjustments are essential for improving estimates in settings with incomplete surveillance, they inherently rely on assumptions that may introduce uncertainty. Diagnostic and reporting challenges, particularly missed infections among individuals who do not seek care or undergo testing, may still lead to underestimation of true dengue incidence. However, sensitivity analyses for these adjustments indicated that the overall distribution of cases were largely consistent, suggesting that the principal findings were robust to reasonable variation in these assumptions.

Dengue incidence is rising globally [27], and Pakistan reflects this trend with an observed rise in cases. While a notable upward trend in dengue incidence was observed nationwide, the data suggests that the disease maintained a baseline endemic state across all time periods. Other studies similarly report a rise in dengue cases, with global incidence increasing from 30.67 million in 1990 to 56.88 million in 2019 [28]. The increase in dengue burden has been observed across all provinces, which may be attributed to factors such as population growth, aging, urbanization, climate change and increased human mobility [29].

Furthermore, our study reveals a geographical disparity in dengue incidence rates, with Sindh emerging as the most affected region, exhibiting consistently high dengue incidence rates from 2012 to 2022, across all age groups. Punjab also demonstrates moderate to high incidence rates, with marked increases. The province of Sindh is predominately hot, humid, and dry, and most affected by rains and flooding, which may create conditions conducive to higher dengue transmission [9,30]. Similarly, Punjab also experiences hot summers and heavy monsoon precipitation and presents an environment that could support dengue transmission during post-monsoon (September-November) [30]. Bostan et al. reported that upper, central and lower Punjab including Lahore, Muzaffargarh, Multan, Rahim Yar Khan, and other areas were at high risk, while Karachi, Ghotki, and some cities of interior Sindh were at high and medium risk. In contrast, the regions of KPK and Baluchistan were categorized as medium risk or low risk [31]. Additionally, districts of Karachi and Hyderabad in Sindh exhibit the highest dengue incidence, likely due to urbanization and migration [31,32].

According to the data from the Pakistan National Institute of Health, there were approximately 22,938 reported cases of dengue fever in 2017, over 3,200 cases in 2018, and 24,547 cases in 2019. There are 25,932 reported confirmed cases post 2022 floods in the country. To our understanding, these numbers underrepresent the dengue incidence in the country as our three-lab and 6% health service utility-based analysis showed higher values (Table 1) across different subnational locations in the country. This highlights a pressing need for a nationwide surveillance system to better understand the landscape of the disease and analyze the geographic and temporal burden. There is an evident gap in the availability of high-quality granular data that can inform targeted interventions and drive policy decision making.

Dengue remains a public health challenge that affects diverse demographic segments across the country. In our study, the increase in dengue cases was observed across all age groups. We found that the 15–49 age group consistently exhibited the highest incidence rates, underscoring the vulnerability of working-age adults to dengue infections [33]. Other studies have reported similar findings, showing that dengue fever has the highest impact on adults aged 16–60 years, with a lower incidence among older adults (≥60 years) [34]. The higher infection rates in this group may be attributed to their high mobility, including time spent in schools, workplaces, and frequent travel to endemic countries [34]. Our study also found that males constituted a higher proportion of dengue cases compared to females, a pattern consistent across different age groups. One study suggested that the male-female disparity could be due to the differences in healthcare-seeking behavior, with adult men more likely to seek healthcare than adult women, leading to a higher number of reported male cases despite no difference in the underlying incidence rates [35,36].

Pakistan has developed both federal and provincial frameworks for dengue prevention and control. The National Institutes of Health (NIH), Pakistan recommends vector surveillance, inspection of residential areas and hotspots, elimination of breeding sites, improved waste management, and insecticide-based measures such as fogging [37]. At the provincial level, Punjab has established more formalized structures for dengue control, including the Dengue Expert Advisory Group and surveillance platforms that support monitoring, training, and response activities [38]. However, existing efforts often depend on the local burden of reported cases and vary across provinces and outbreak periods, with implementation frequently occurring on an ad-hoc basis [38–40]. With the increase in dengue burden, these efforts should be coordinated, sustained, and operationalized more consistently and proactively. High-incidence districts would benefit from strengthened routine district-level vector surveillance, routine identification and removal of breeding sites, intersectoral coordination with municipal services, and timely insecticide-based control of adult mosquitoes during outbreaks when indicated [41,42]. To further curb the spread of dengue, policy makers should also consider well-established preventive strategies such as enhanced water, sanitation, and hygiene (WASH) facilities. To mitigate the risk during floods and heavy monsoon rains, efforts should focus on preventing water stagnation by improving drainage systems and implementing large-scale water evacuation measures in flood-affected areas [43]. Additionally, integrating dengue vector control with other vector-borne disease control programs may further enhance the feasibility and sustainability of these interventions [41].

A dengue vaccine offers a long-term, sustainable solution to reduce the growing burden of the disease. Currently, there are no dengue vaccines available in Pakistan, despite the increasing number of cases and documented outbreaks across various regions in the country [44]. Our findings underscore an urgent need for policymakers to introduce a dengue vaccine to complement existing preventive strategies and reduce the overall incidence of the disease

Despite providing valuable insights, this study has several limitations that require acknowledgement. Firstly, the data used in the analysis was derived from laboratory-confirmed cases that would not have captured all dengue infections, particularly those that are asymptomatic or not reported. Secondly, the estimates were influenced by the presence and distribution of laboratory collection units. A lower incidence rate in certain regions may not necessarily reflect a lower number of infections but could be due to limited utilization of lab facilities. Lastly, the study does not account for other potential factors influencing dengue incidence, such as measures of variables (temperature, rainfall, relative humidity, etc.) resulting from climate change, anthropogenic factors like trends in urbanization and migration, and changes in vector populations, which could provide a more comprehensive understanding of the observed trends.

## Conclusion

This study provides a comprehensive estimation of the temporal and spatial trends of dengue in Pakistan over an 11-year period. Our findings reveal a substantial increase in dengue incidence rate, particularly among individuals aged 15–49 years and in the province of Sindh. While our analysis provides valuable insights, it is important to acknowledge that this study relied on laboratory-confirmed cases, which may underestimate the true burden of dengue due to asymptomatic infections and underreporting. To address these limitations and inform effective public health strategies, future research should incorporate serological surveys to capture asymptomatic cases and conduct detailed ecological studies to assess the influence of environmental factors on dengue transmission.

## Supporting information

S1 Table STROBE checklist. (DOCX)

S2 Table Data sharing template shared with partnering labs. (DOCX)

S1 Fig Population coverage by distance to nearest clinic within district across four provinces. Mean percentage of the district population residing within a given distance (0–20 km) of at least one collection unit located in the same district, shown separately for each province. Districts are stratified by the number of collection units per district (0, 1–4, 5–9, ≥ 10). Shaded bands represent the 2.5th–97.5th percentile range across districts within each collection unit–density category.
(TIF)

S2 Fig Collection Units of Partnering Labs Across Pakistan. Shape file source: World Food Programme SDI, URL: https://data.humdata.org/dataset/cod-em-pak under a CC BY license.
(TIF)

S3 Fig Dengue Testing Across Pakistan. Shape file source: World Food Programme SDI, URL: https://data.humdata.org/dataset/cod-em-pak under a CC BY license.
(TIF)

S4 Fig a) Distribution of Dengue Cases across Age Groups b) Cumulative Incidence Per 1000 across Age Groups. (TIF)

S5 Fig Age and Sex Distribution of Dengue Cases (2012–2022). (TIF)

S6 Fig Province-wise monthly trends in laboratory-confirmed dengue cases in Pakistan, January 2012–December 2022. (TIF)

S7 Fig Baseline Trend of Dengue Cases in Pakistan. (TIF)

S8 Fig Sensitivity Analysis of Dengue Incidence Rate per 1000 in Sindh, 2022. (TIF)

S9 Fig Dengue Incidence Across Different Age Groups in Pakistan for the Years a) 2012 and b)2022. Shape file source: World Food Programme SDI, URL: https://data.humdata.org/dataset/cod-em-pak under a CC BY license.
(ZIP)

S3 Table Incidence Rate of Dengue across Districts. (DOCX)
