# Supplementary material for: Incidence of Dengue Fever in Pakistan
Source: PLoS One. 2026 Jul 2;21(7):e0352938. doi: 10.1371/journal.pone.0352938 (PMC13327124; doi:10.1371/journal.pone.0352938)
Supplement: S2 Table — (DOCX) [file pone.0352938.s002.docx]

**S2 Table:** Data sharing template shared with partnering labs

| **Variable** | **Description** |
| --- | --- |
| 1. Time Period | Jan 2012-Dec 2022 |
| 1. PATIENTUNITNUMBER | Deidentified Unique identifier for each patient |
| 1. Age | Numeric |
| 1. VISITID | Unique identifier for each lab visit. |
| 1. Sex: | 1. Male 2. Female |
| Laboratory Investigations |  |
| 1. DENGUE IgM ANTIBODY | 1. positive  2. negative |
| 1. Dengue NS1 Antigen detection (Qualitative) | 1. positive  2. negative |
| 1. ORDEREDDATETIME | Date and time when the test was ordered. |
| 1. RESULTDATETIME | Date and time when the test result was available. |
| Collection Point |  |
| 1. NAME | Name of the collection unit. |
| 1. PROVINCE | Province where the collection unit is located. |
| 1. CITY | City where the collection unit is located. |
| 1. DISTRICT | District where the collection unit is located. |
| 1. LATITUDE | Latitude coordinate of the collection unit. |
| 1. LONGITUDE | Longitude coordinate of the collection unit. |
| Outcome |  |
| 1. Dengue Outcome (Laboratory Result) | DETECTED/NOT DETECTED |
