# Supplementary figures and images for: Incidence of Dengue Fever in Pakistan
Source: PLoS One. 2026 Jul 2;21(7):e0352938. doi: 10.1371/journal.pone.0352938 (PMC13327124; doi:10.1371/journal.pone.0352938)

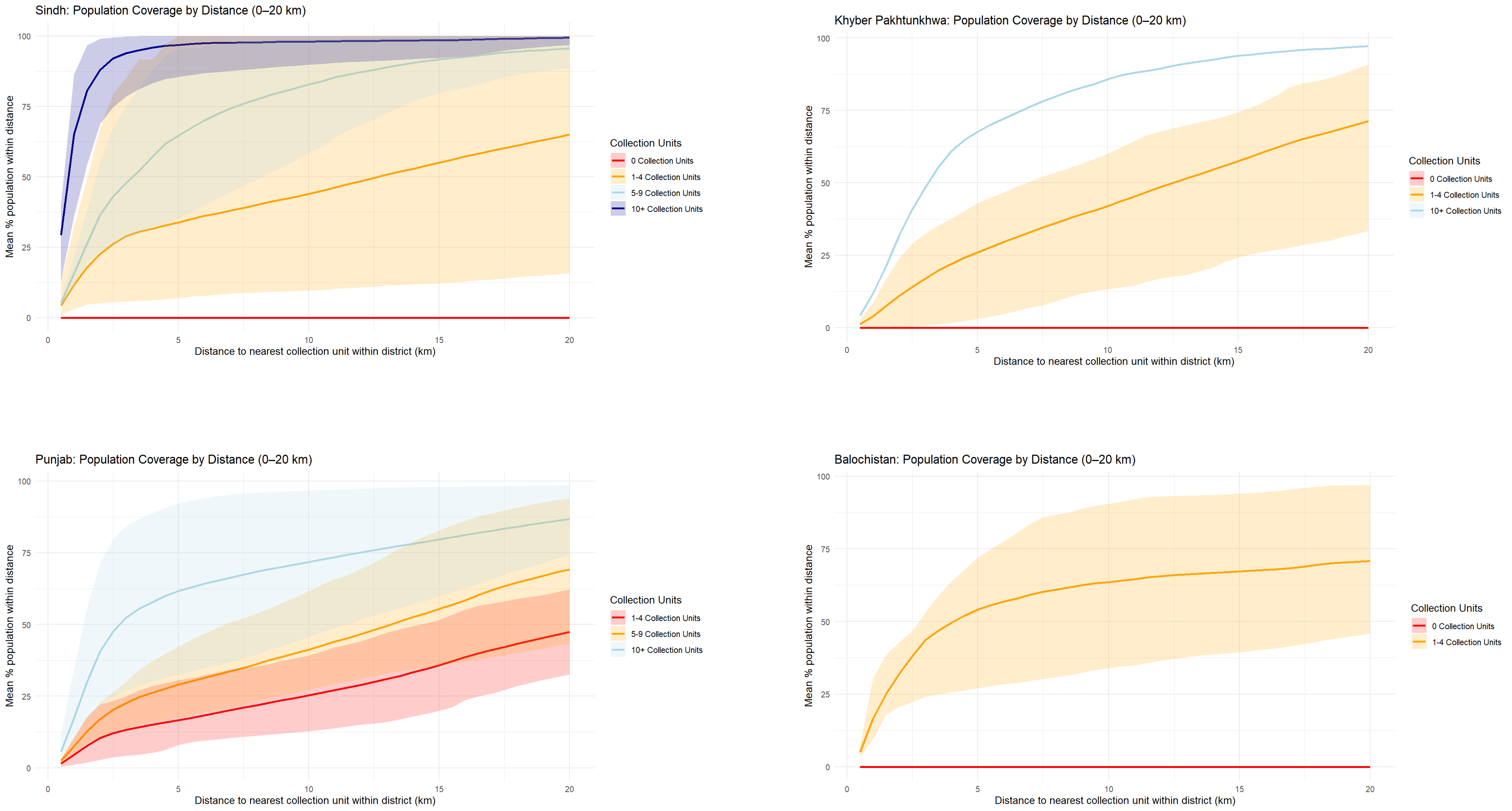

Supplement: S1 Fig — Mean percentage of the district population residing within a given distance (0–20 km) of at least one collection unit located in the same district, shown separately for each province. Districts are stratified by the number of collection units per district (0, 1–4, 5–9, ≥ 10). Shaded bands represent the 2.5th–97.5th percentile range across districts within each collection unit–density category. (TIF) [file pone.0352938.s003.tif]

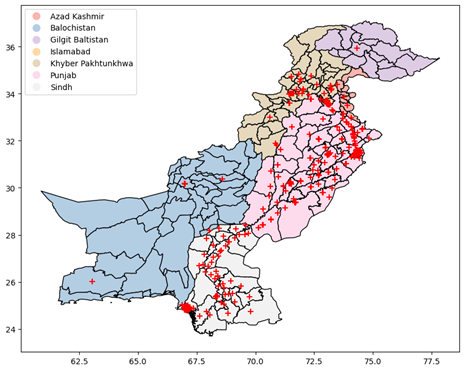

Supplement: S2 Fig — Shape file source: World Food Programme SDI, URL: https://data.humdata.org/dataset/cod-em-pak under a CC BY license. (TIF) [file pone.0352938.s004.tif]

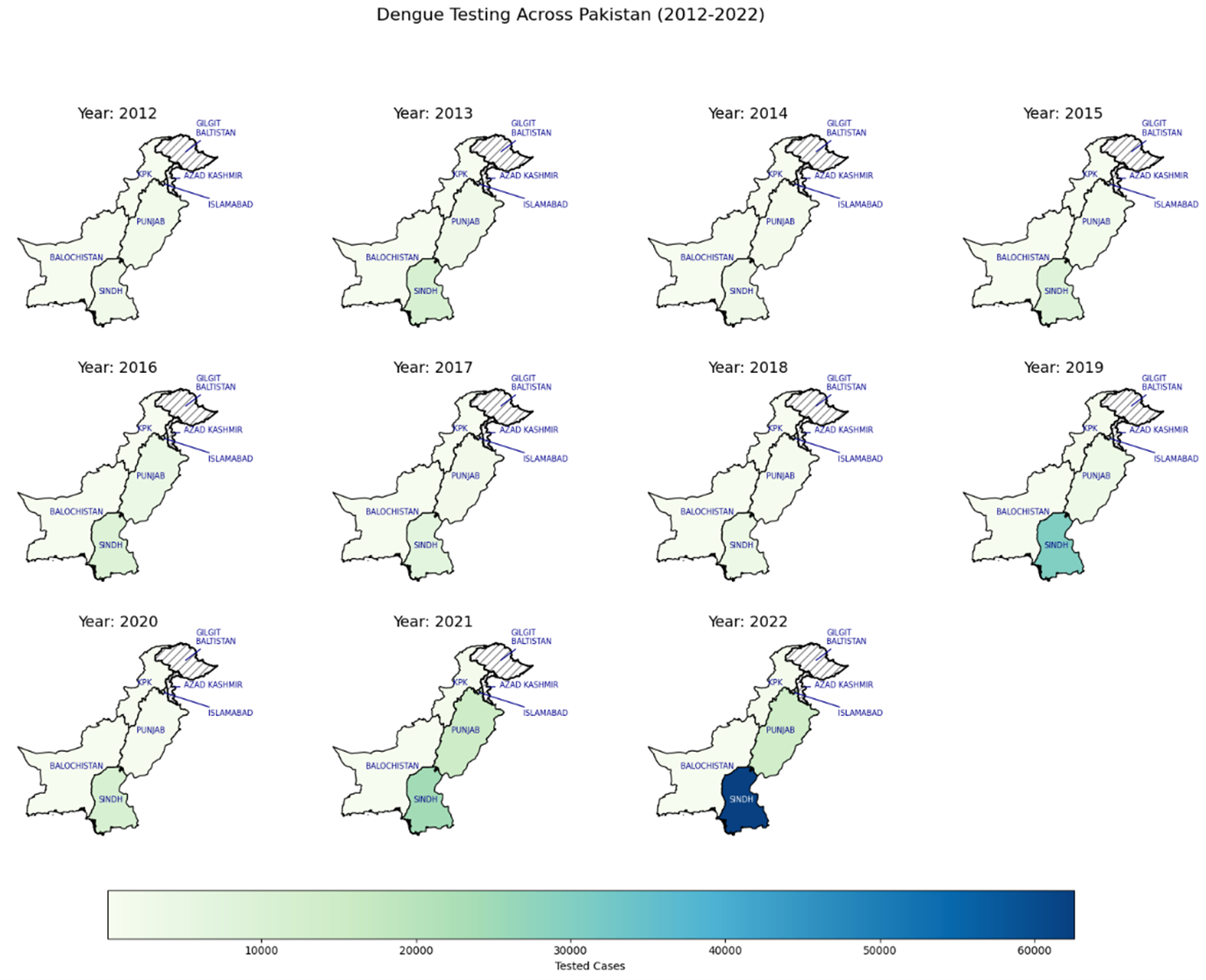

Supplement: S3 Fig — Shape file source: World Food Programme SDI, URL: https://data.humdata.org/dataset/cod-em-pak under a CC BY license. (TIF) [file pone.0352938.s005.tif]

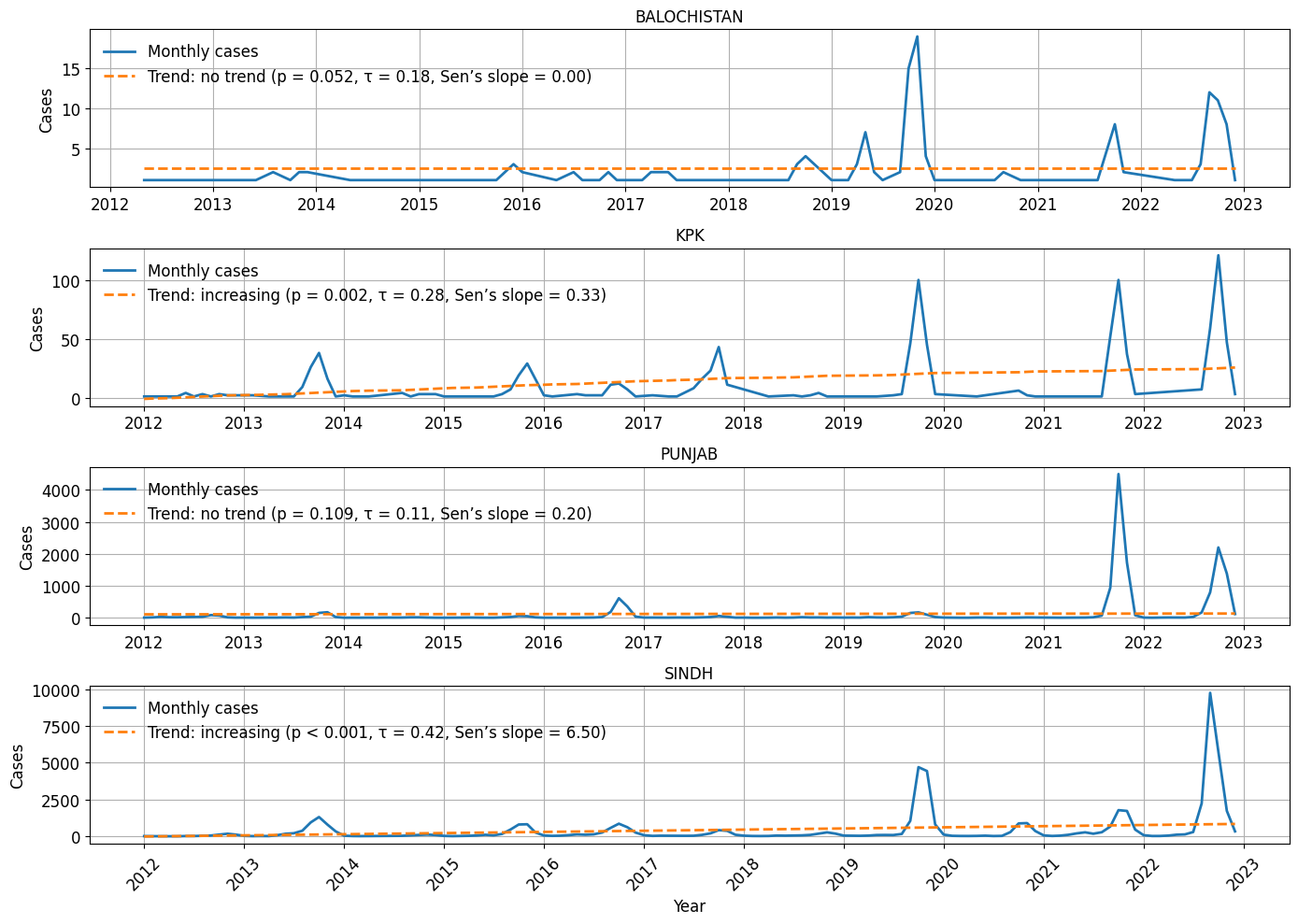

Supplement: S4 Fig — (TIF) [file pone.0352938.s006.tif]

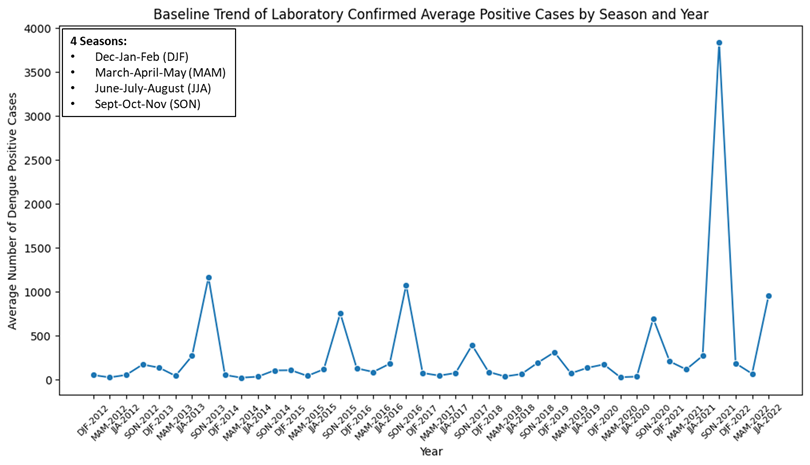

Supplement: S5 Fig — (TIF) [file pone.0352938.s007.tif]

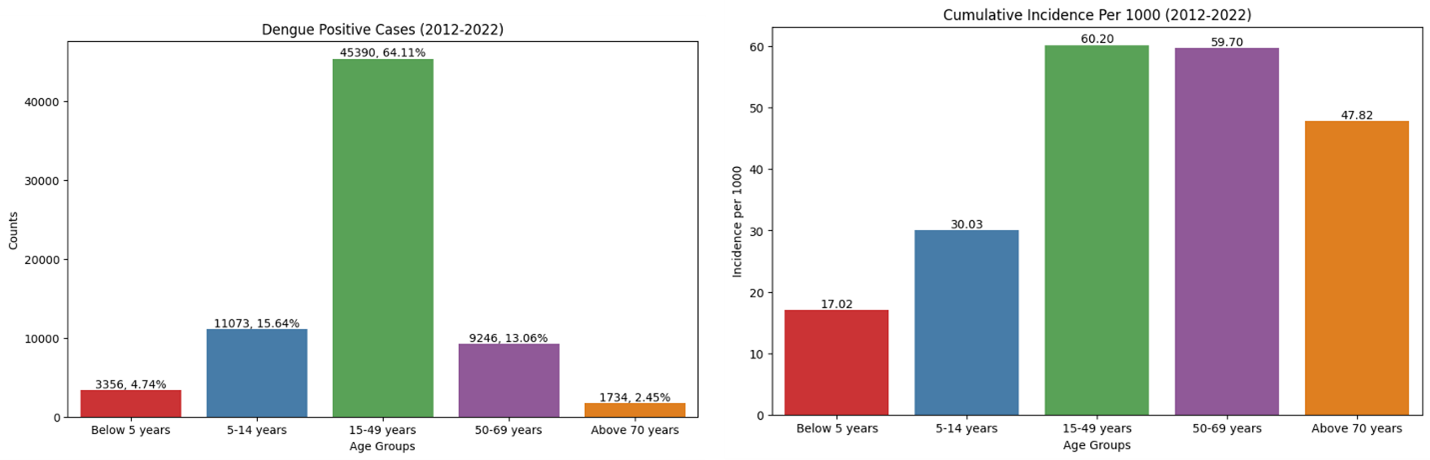

Supplement: S6 Fig — (TIF) [file pone.0352938.s008.tif]

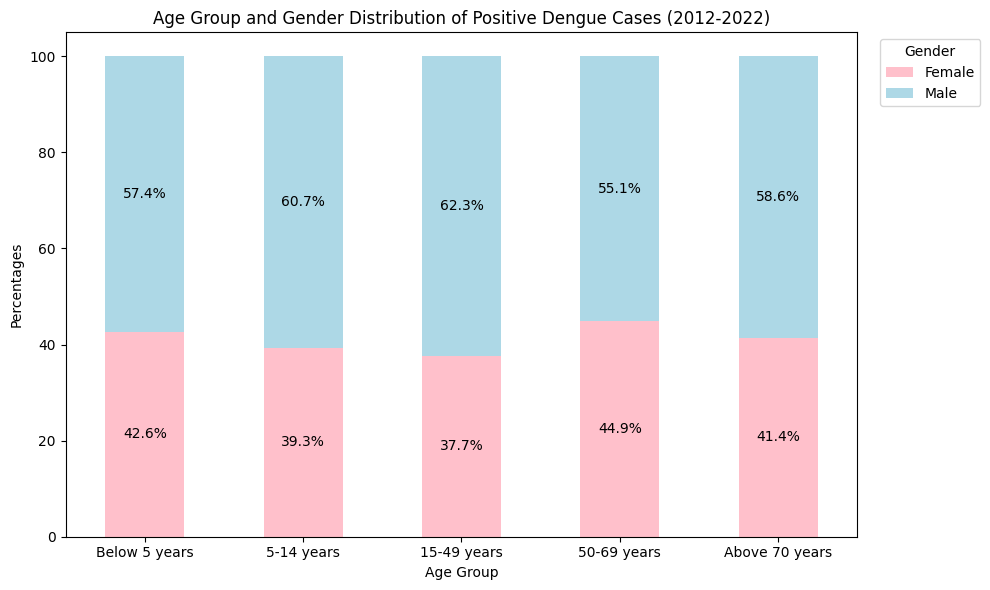

Supplement: S7 Fig — (TIF) [file pone.0352938.s009.tif]

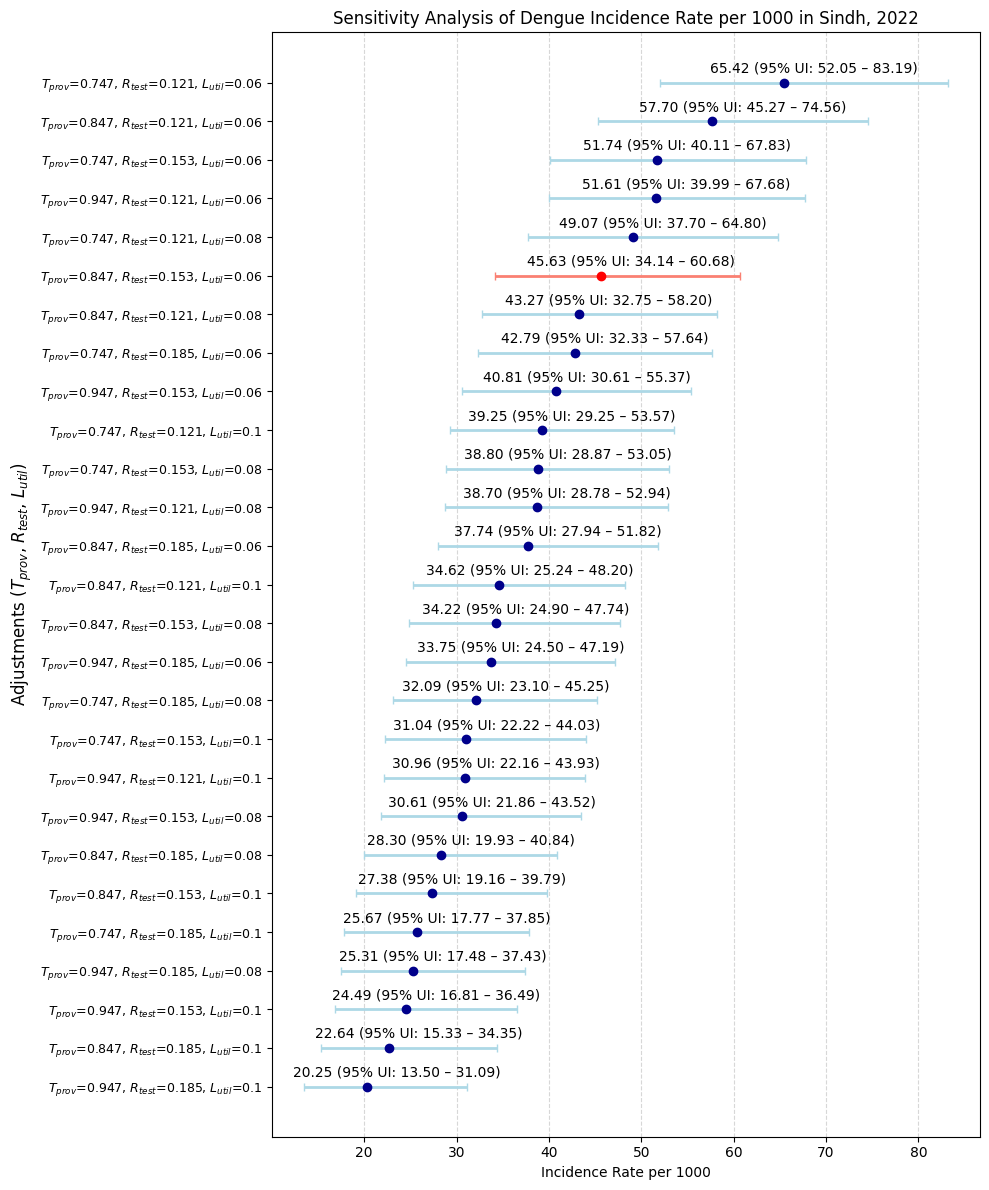

Supplement: S8 Fig — (TIF) [file pone.0352938.s010.tif]

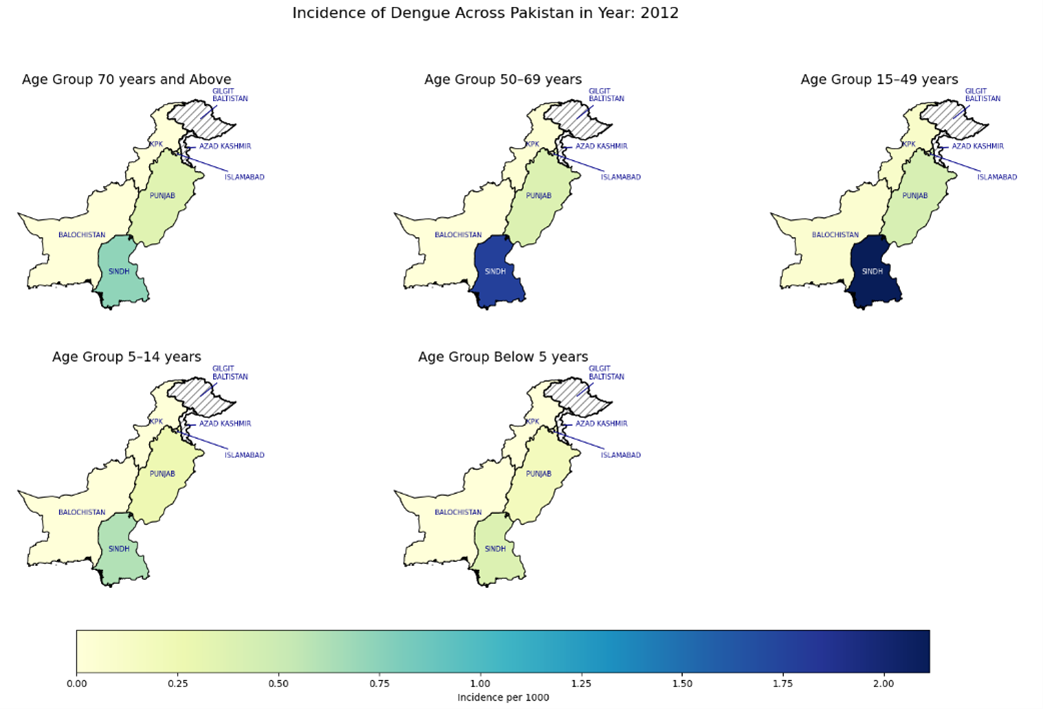

Supplement: S9 Fig — Shape file source: World Food Programme SDI, URL: https://data.humdata.org/dataset/cod-em-pak under a CC BY license. (ZIP) [file pone.0352938.s011.zip › SupplementalFigure8/SupplementalFigure8a.tif]

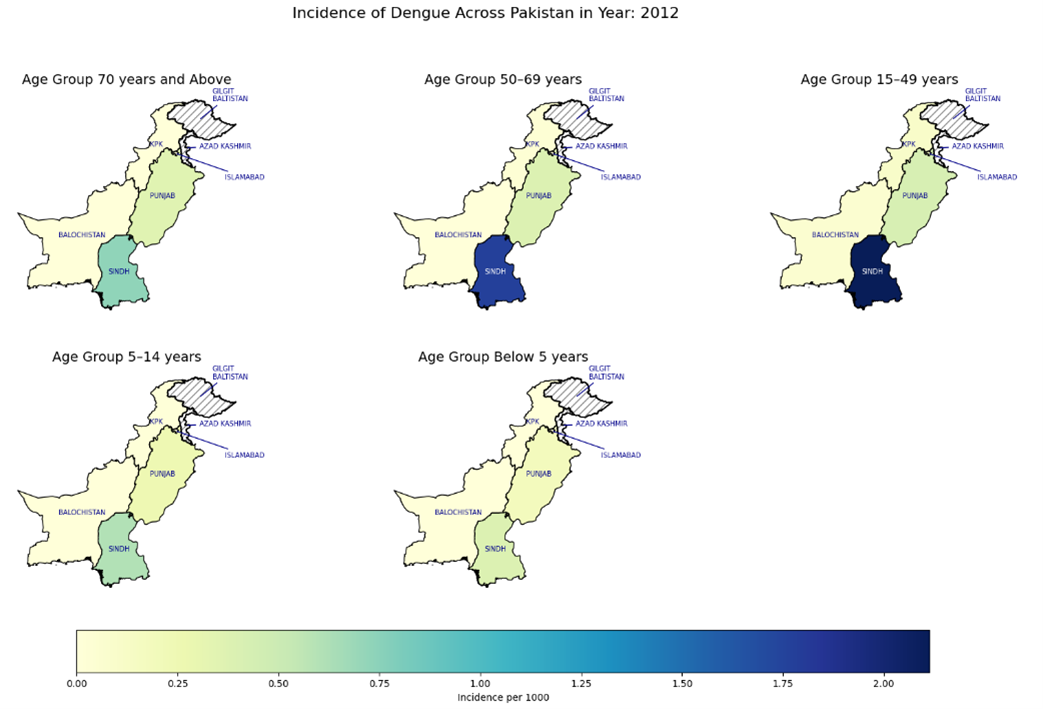

Supplement: S9 Fig — Shape file source: World Food Programme SDI, URL: https://data.humdata.org/dataset/cod-em-pak under a CC BY license. (ZIP) [file pone.0352938.s011.zip › SupplementalFigure8/SupplementalFigure8a.tif.png]

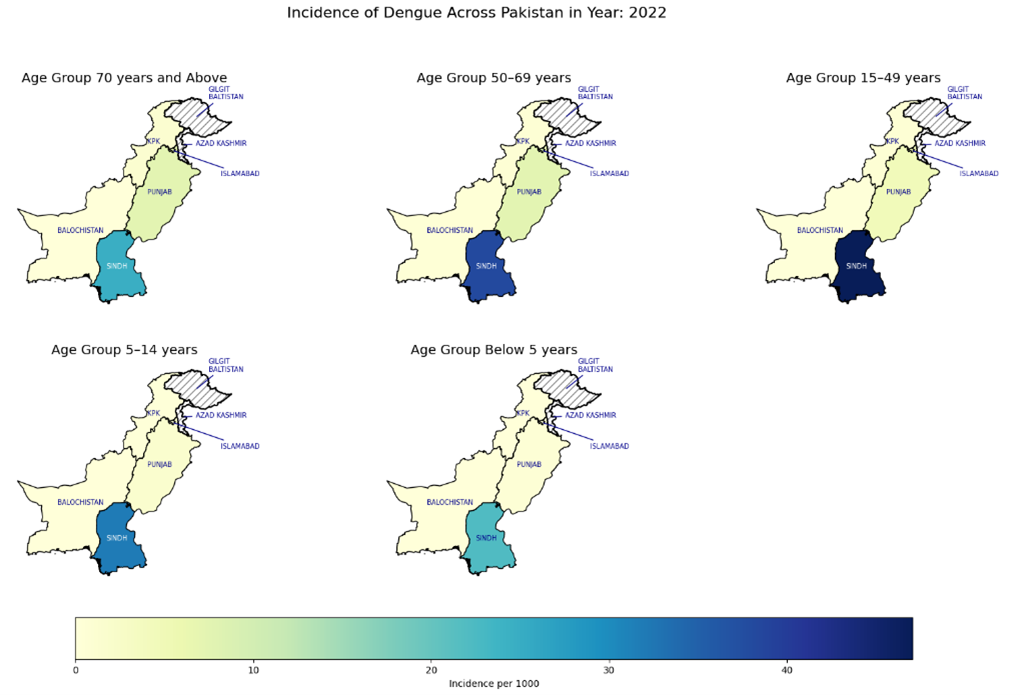

Supplement: S9 Fig — Shape file source: World Food Programme SDI, URL: https://data.humdata.org/dataset/cod-em-pak under a CC BY license. (ZIP) [file pone.0352938.s011.zip › SupplementalFigure8/SupplementalFigure8b.tif]
