# Supplementary material for: Incidence of Dengue Fever in Pakistan
Source: PLoS One. 2026 Jul 2;21(7):e0352938. doi: 10.1371/journal.pone.0352938 (PMC13327124; doi:10.1371/journal.pone.0352938)
Supplement: S3 Table — (DOCX) [file pone.0352938.s012.docx]

**S3 Table: Incidence Rate of Dengue across Districts**

| **DISTRICT** | **YEAR** | **PROVINCE** | **Incidence Rate Per 1000** | **lower_UI** | **upper_UI** |
| --- | --- | --- | --- | --- | --- |
| ABBOTTABAD | 2012 | KPK | 0.505817 | 0.12317 | 4.529295 |
| ATTOCK | 2012 | PUNJAB | 2.525253 | 0.86827 | 7.76957 |
| BADIN | 2012 | SINDH | 5.050505 | 2.369683 | 11.59896 |
| BAHAWALNAGAR | 2012 | PUNJAB | 0.046692 | 0.024436 | 3.542664 |
| BAHAWALPUR | 2012 | PUNJAB | 0.038191 | 0.023731 | 3.791573 |
| CHAKWAL | 2012 | PUNJAB | 2.680965 | 0.998887 | 8.427041 |
| CHINIOT | 2012 | PUNJAB | 0 | 0.032172 | 3.727125 |
| DADU | 2012 | SINDH | 0.97561 | 0.245894 | 5.736849 |
| DERA GHAZI KHAN | 2012 | PUNJAB | 1.693002 | 0.48239 | 6.384307 |
| D. I. KHAN | 2012 | KPK | 1.95122 | 0.622491 | 7.722569 |
| FAISALABAD | 2012 | PUNJAB | 0.7235 | 0.166062 | 4.938236 |
| GHOTKI | 2012 | SINDH | 18.89764 | 12.21773 | 28.8756 |
| GUJRANWALA | 2012 | PUNJAB | 0.445422 | 0.115534 | 4.52344 |
| GUJRAT | 2012 | PUNJAB | 0.298151 | 0.05198 | 4.162091 |
| HAFIZABAD | 2012 | PUNJAB | 0 | 0.017678 | 3.760661 |
| HARIPUR | 2012 | KPK | 1.652893 | 0.452246 | 6.342917 |
| HYDERABAD | 2012 | SINDH | 1.276895 | 0.311397 | 6.276691 |
| JACOBABAD | 2012 | SINDH | 0 | 0.027353 | 3.956588 |
| JHANG | 2012 | PUNJAB | 0 | 0.027518 | 3.842921 |
| JHELUM | 2012 | PUNJAB | 0 | 0.028016 | 3.468302 |
| CENTRAL KARACHI | 2012 | SINDH | 2.257232 | 0.758893 | 7.624856 |
| EAST KARACHI | 2012 | SINDH | 11.21688 | 6.259053 | 19.99517 |
| SOUTH KARACHI | 2012 | SINDH | 15.98052 | 9.845781 | 25.36321 |
| WEST KARACHI | 2012 | SINDH | 0.1496 | 0.046053 | 3.874068 |
| KASHMORE | 2012 | SINDH | 0 | 0.024568 | 3.940938 |
| KHAIRPUR | 2012 | SINDH | 0.71582 | 0.164074 | 4.808032 |
| KHANEWAL | 2012 | PUNJAB | 0.692521 | 0.148165 | 5.365926 |
| KORANGI KARACHI | 2012 | SINDH | 1.328977 | 0.296856 | 5.478555 |
| LAHORE | 2012 | PUNJAB | 2.706342 | 0.938927 | 8.083235 |
| LARKANA | 2012 | SINDH | 1.447178 | 0.373681 | 6.291047 |
| LASBELA | 2012 | BALOCHISTAN | 0 | 0.028572 | 3.801449 |
| LEIAH | 2012 | PUNJAB | 7.782101 | 3.9077 | 15.13208 |
| LORALAI | 2012 | BALOCHISTAN | 0 | 0.028847 | 3.9872 |
| LOWER DIR | 2012 | KPK | 0 | 0.02764 | 3.681676 |
| MALIR KARACHI | 2012 | SINDH | 1.240599 | 0.288657 | 5.997962 |
| MANDI BAHAUDDIN | 2012 | PUNJAB | 0 | 0.020609 | 3.889351 |
| MANSEHRA | 2012 | KPK | 0 | 0.027185 | 3.381857 |
| MARDAN | 2012 | KPK | 0 | 0.023164 | 3.748876 |
| MATIARI | 2012 | SINDH | 0 | 0.015448 | 3.71935 |
| MIANWALI | 2012 | PUNJAB | 0 | 0.019546 | 3.554556 |
| MIRPUR KHAS | 2012 | SINDH | 2.835539 | 1.066641 | 8.613644 |
| MULTAN | 2012 | PUNJAB | 0.295788 | 0.083312 | 4.376548 |
| NAUSHAHRO FEROZE | 2012 | SINDH | 0 | 0.021083 | 3.467146 |
| NOWSHERA | 2012 | KPK | 2.820874 | 1.032829 | 8.262206 |
| OKARA | 2012 | PUNJAB | 0 | 0.027578 | 3.50931 |
| PAKPATTAN | 2012 | PUNJAB | 1.362398 | 0.343283 | 6.380975 |
| PESHAWAR | 2012 | KPK | 0.497121 | 0.087172 | 4.432515 |
| QUETTA | 2012 | BALOCHISTAN | 2.33827 | 0.750435 | 7.926466 |
| RAHIM YAR KHAN | 2012 | PUNJAB | 0.058457 | 0.032475 | 3.859125 |
| RAJANPUR | 2012 | PUNJAB | 0 | 0.026951 | 3.741566 |
| RAWALPINDI | 2012 | PUNJAB | 0 | 0.024224 | 3.459989 |
| SAHIWAL | 2012 | PUNJAB | 0.273254 | 0.077564 | 4.410695 |
| SANGHAR | 2012 | SINDH | 0 | 0.016913 | 3.615054 |
| SARGODHA | 2012 | PUNJAB | 0.149142 | 0.034073 | 4.043145 |
| SHAHEED BENAZIR ABAD | 2012 | SINDH | 0.086957 | 0.034667 | 3.660756 |
| SHEIKHUPURA | 2012 | PUNJAB | 0.121684 | 0.035753 | 3.932241 |
| SHIKARPUR | 2012 | SINDH | 1.936108 | 0.651506 | 6.846438 |
| SIALKOT | 2012 | PUNJAB | 0.071212 | 0.042652 | 4.100645 |
| SUKKUR | 2012 | SINDH | 3.479125 | 1.381506 | 9.049098 |
| SWABI | 2012 | KPK | 0 | 0.020772 | 3.994841 |
| TANDO ALLAHYAR | 2012 | SINDH | 0 | 0.025662 | 3.802194 |
| TANDO MUHAMMAD KHAN | 2012 | SINDH | 0 | 0.024766 | 3.438555 |
| THATTA | 2012 | SINDH | 0 | 0.028003 | 3.42531 |
| TOBA TEK SINGH | 2012 | PUNJAB | 0 | 0.020755 | 3.944854 |
| UMER KOT | 2012 | SINDH | 0 | 0.024195 | 3.650745 |
| ABBOTTABAD | 2013 | KPK | 2.97619 | 0.972455 | 8.290402 |
| ATTOCK | 2013 | PUNJAB | 7.444169 | 3.87755 | 14.60029 |
| BADIN | 2013 | SINDH | 4.938272 | 2.091854 | 11.60457 |
| BAHAWALNAGAR | 2013 | PUNJAB | 0 | 0.030581 | 3.606809 |
| BAHAWALPUR | 2013 | PUNJAB | 0.187308 | 0.046816 | 4.285532 |
| BANNU | 2013 | KPK | 0 | 0.025391 | 3.807709 |
| CHAKWAL | 2013 | PUNJAB | 0 | 0.034349 | 3.478954 |
| CHINIOT | 2013 | PUNJAB | 0 | 0.029525 | 3.650529 |
| DADU | 2013 | SINDH | 0 | 0.024553 | 3.615102 |
| DERA GHAZI KHAN | 2013 | PUNJAB | 1.651073 | 0.46148 | 6.368639 |
| D. I. KHAN | 2013 | KPK | 2.843602 | 1.061891 | 8.40676 |
| FAISALABAD | 2013 | PUNJAB | 0.29473 | 0.067319 | 4.202732 |
| GHOTKI | 2013 | SINDH | 18.46154 | 11.29838 | 28.09345 |
| GUJRANWALA | 2013 | PUNJAB | 0.246002 | 0.076749 | 4.251058 |
| GUJRAT | 2013 | PUNJAB | 0.097962 | 0.033014 | 4.30832 |
| HARIPUR | 2013 | KPK | 1.623377 | 0.474221 | 6.707351 |
| HYDERABAD | 2013 | SINDH | 53.10012 | 40.35803 | 69.10877 |
| JACOBABAD | 2013 | SINDH | 3.31675 | 1.403663 | 9.582857 |
| JHANG | 2013 | PUNJAB | 0 | 0.023369 | 3.543567 |
| JHELUM | 2013 | PUNJAB | 0 | 0.029063 | 3.945819 |
| KAMBAR SHAHDAD KOT | 2013 | SINDH | 0 | 0.033306 | 3.810383 |
| CENTRAL KARACHI | 2013 | SINDH | 36.33966 | 25.98513 | 48.55753 |
| EAST KARACHI | 2013 | SINDH | 67.38262 | 53.87337 | 84.80642 |
| SOUTH KARACHI | 2013 | SINDH | 50.58806 | 38.51058 | 64.85658 |
| WEST KARACHI | 2013 | SINDH | 5.206247 | 2.466512 | 11.74077 |
| KASHMORE | 2013 | SINDH | 1.451379 | 0.450336 | 6.421118 |
| KECH | 2013 | BALOCHISTAN | 18.34862 | 11.48514 | 29.44408 |
| KHAIRPUR | 2013 | SINDH | 1.402525 | 0.424122 | 6.009103 |
| KHANEWAL | 2013 | PUNJAB | 0.681199 | 0.179518 | 5.086292 |
| KORANGI KARACHI | 2013 | SINDH | 9.752926 | 4.935223 | 17.65612 |
| LAHORE | 2013 | PUNJAB | 3.687055 | 1.40404 | 9.729352 |
| LARKANA | 2013 | SINDH | 4.257332 | 1.776697 | 10.44018 |
| LASBELA | 2013 | BALOCHISTAN | 64.51613 | 49.74925 | 80.34211 |
| LEIAH | 2013 | PUNJAB | 1.90295 | 0.652074 | 6.639807 |
| MALIR KARACHI | 2013 | SINDH | 8.1142 | 4.367311 | 16.11849 |
| MANDI BAHAUDDIN | 2013 | PUNJAB | 1.892148 | 0.567346 | 6.928727 |
| MANSEHRA | 2013 | KPK | 2.97619 | 1.06532 | 8.417455 |
| MARDAN | 2013 | KPK | 3.755869 | 1.442012 | 9.870803 |
| MATIARI | 2013 | SINDH | 8.87574 | 4.614593 | 17.02284 |
| MIANWALI | 2013 | PUNJAB | 2.597403 | 0.84044 | 8.159723 |
| MIRPUR KHAS | 2013 | SINDH | 10.20408 | 5.605266 | 18.52988 |
| MULTAN | 2013 | PUNJAB | 0.261028 | 0.055911 | 4.109482 |
| NANKANA SAHIB | 2013 | PUNJAB | 0 | 0.024432 | 3.302752 |
| NAUSHAHRO FEROZE | 2013 | SINDH | 1.879699 | 0.606502 | 6.672631 |
| NOWSHERA | 2013 | KPK | 1.375516 | 0.381406 | 5.766086 |
| OKARA | 2013 | PUNJAB | 1.265823 | 0.304291 | 5.966694 |
| PAKPATTAN | 2013 | PUNJAB | 0 | 0.024051 | 3.790164 |
| PESHAWAR | 2013 | KPK | 2.689359 | 1.003839 | 8.022658 |
| QUETTA | 2013 | BALOCHISTAN | 5.235602 | 2.166295 | 12.29141 |
| RAHIM YAR KHAN | 2013 | PUNJAB | 0.08596 | 0.033562 | 3.854616 |
| RAJANPUR | 2013 | PUNJAB | 0 | 0.021504 | 3.874633 |
| RAWALPINDI | 2013 | PUNJAB | 0.616285 | 0.113673 | 4.765992 |
| SAHIWAL | 2013 | PUNJAB | 0.215355 | 0.066345 | 4.381246 |
| SANGHAR | 2013 | SINDH | 4.232804 | 1.720963 | 10.03595 |
| SARGODHA | 2013 | PUNJAB | 0.036704 | 0.031594 | 3.652164 |
| SHAHEED BENAZIR ABAD | 2013 | SINDH | 0.085404 | 0.031347 | 3.509637 |
| SHEIKHUPURA | 2013 | PUNJAB | 0.159096 | 0.031393 | 4.158852 |
| SHIKARPUR | 2013 | SINDH | 2.857143 | 0.92935 | 8.555546 |
| SIALKOT | 2013 | PUNJAB | 0.140017 | 0.045318 | 3.938713 |
| SUKKUR | 2013 | SINDH | 6.322957 | 2.91637 | 13.20475 |
| SWABI | 2013 | KPK | 0 | 0.021924 | 3.92901 |
| SWAT | 2013 | KPK | 13.81693 | 7.906421 | 23.29842 |
| TANDO ALLAHYAR | 2013 | SINDH | 3.10559 | 1.183858 | 8.887617 |
| TANDO MUHAMMAD KHAN | 2013 | SINDH | 3.184713 | 1.264469 | 8.855625 |
| THARPARKAR | 2013 | SINDH | 58.82353 | 46.99133 | 74.964 |
| THATTA | 2013 | SINDH | 7.978723 | 4.333963 | 15.55738 |
| TOBA TEK SINGH | 2013 | PUNJAB | 0 | 0.023943 | 3.859189 |
| UMER KOT | 2013 | SINDH | 23.64865 | 15.90675 | 35.86093 |
| VEHARI | 2013 | PUNJAB | 0 | 0.026892 | 3.894981 |
| ABBOTTABAD | 2014 | KPK | 0 | 0.022515 | 3.75824 |
| ATTOCK | 2014 | PUNJAB | 0 | 0.028067 | 3.57138 |
| BADIN | 2014 | SINDH | 0 | 0.023396 | 3.452288 |
| BAHAWALNAGAR | 2014 | PUNJAB | 0 | 0.025441 | 3.98595 |
| BAHAWALPUR | 2014 | PUNJAB | 0 | 0.026013 | 4.080394 |
| CHAKWAL | 2014 | PUNJAB | 0 | 0.025725 | 3.909036 |
| CHINIOT | 2014 | PUNJAB | 0.856898 | 0.152755 | 5.120968 |
| DADU | 2014 | SINDH | 0.94518 | 0.222612 | 5.266086 |
| DERA GHAZI KHAN | 2014 | PUNJAB | 1.074114 | 0.290887 | 6.29103 |
| D. I. KHAN | 2014 | KPK | 0.922509 | 0.206556 | 5.452717 |
| FAISALABAD | 2014 | PUNJAB | 0.136307 | 0.042627 | 4.030178 |
| GHOTKI | 2014 | SINDH | 1.501502 | 0.361913 | 6.357112 |
| GUJRANWALA | 2014 | PUNJAB | 0.107385 | 0.036701 | 4.081341 |
| GUJRAT | 2014 | PUNJAB | 0 | 0.023849 | 3.546115 |
| HAFIZABAD | 2014 | PUNJAB | 0 | 0.026526 | 3.492499 |
| HARIPUR | 2014 | KPK | 1.597444 | 0.424488 | 6.514858 |
| HYDERABAD | 2014 | SINDH | 1.477833 | 0.420762 | 6.463 |
| JACOBABAD | 2014 | SINDH | 1.633987 | 0.436907 | 6.528638 |
| JHANG | 2014 | PUNJAB | 0 | 0.017962 | 3.479623 |
| JHELUM | 2014 | PUNJAB | 0 | 0.026092 | 3.835308 |
| CENTRAL KARACHI | 2014 | SINDH | 3.727657 | 1.53531 | 9.498488 |
| EAST KARACHI | 2014 | SINDH | 7.765482 | 3.661189 | 15.67493 |
| SOUTH KARACHI | 2014 | SINDH | 14.49167 | 8.665638 | 24.18815 |
| WEST KARACHI | 2014 | SINDH | 0.212804 | 0.034842 | 4.413149 |
| KASHMORE | 2014 | SINDH | 1.422475 | 0.339107 | 6.021669 |
| KECH | 2014 | BALOCHISTAN | 0 | 0.026704 | 3.262865 |
| KHAIRPUR | 2014 | SINDH | 1.37457 | 0.313676 | 5.801298 |
| KHANEWAL | 2014 | PUNJAB | 0 | 0.016984 | 3.49366 |
| KORANGI KARACHI | 2014 | SINDH | 1.697433 | 0.485313 | 6.410424 |
| LAHORE | 2014 | PUNJAB | 0.455856 | 0.091726 | 4.015745 |
| LARKANA | 2014 | SINDH | 1.856148 | 0.613275 | 6.708985 |
| LASBELA | 2014 | BALOCHISTAN | 15.74803 | 9.475881 | 25.70031 |
| LEIAH | 2014 | PUNJAB | 0.931099 | 0.240324 | 5.512996 |
| MALIR KARACHI | 2014 | SINDH | 1.020185 | 0.246824 | 5.934984 |
| MANSEHRA | 2014 | KPK | 0 | 0.023027 | 3.614071 |
| MARDAN | 2014 | KPK | 0.459348 | 0.0838 | 4.812543 |
| MATIARI | 2014 | SINDH | 2.898551 | 1.051695 | 8.516219 |
| MIANWALI | 2014 | PUNJAB | 0 | 0.027853 | 3.600765 |
| MIRPUR KHAS | 2014 | SINDH | 0.910747 | 0.167847 | 5.928054 |
| MULTAN | 2014 | PUNJAB | 0.056897 | 0.0324 | 3.891393 |
| MUZAFFARGARH | 2014 | PUNJAB | 0 | 0.027154 | 3.335959 |
| NANKANA SAHIB | 2014 | PUNJAB | 0 | 0.029146 | 3.576637 |
| NAUSHAHRO FEROZE | 2014 | SINDH | 1.846154 | 0.519884 | 6.91396 |
| NOWSHERA | 2014 | KPK | 0 | 0.021969 | 3.525044 |
| OKARA | 2014 | PUNJAB | 0.62383 | 0.168798 | 5.234863 |
| PESHAWAR | 2014 | KPK | 0.583953 | 0.134609 | 4.958064 |
| QUETTA | 2014 | BALOCHISTAN | 1.437815 | 0.367044 | 6.377069 |
| RAHIM YAR KHAN | 2014 | PUNJAB | 0.0281 | 0.026909 | 3.487778 |
| RAJANPUR | 2014 | PUNJAB | 0 | 0.023372 | 3.422704 |
| RAWALPINDI | 2014 | PUNJAB | 0.050273 | 0.024111 | 3.701072 |
| SAHIWAL | 2014 | PUNJAB | 0 | 0.016824 | 3.057915 |
| SANGHAR | 2014 | SINDH | 0.518672 | 0.124962 | 4.809965 |
| SARGODHA | 2014 | PUNJAB | 0.07228 | 0.037688 | 4.047528 |
| SHAHEED BENAZIR ABAD | 2014 | SINDH | 0.083907 | 0.043082 | 3.600625 |
| SHEIKHUPURA | 2014 | PUNJAB | 0.078034 | 0.038523 | 3.773685 |
| SHIKARPUR | 2014 | SINDH | 0 | 0.016557 | 3.669157 |
| SIALKOT | 2014 | PUNJAB | 0 | 0.027214 | 3.793613 |
| SUKKUR | 2014 | SINDH | 4.285714 | 1.597848 | 10.41702 |
| SWABI | 2014 | KPK | 0 | 0.032308 | 3.87895 |
| TANDO ALLAHYAR | 2014 | SINDH | 0 | 0.021205 | 3.945019 |
| TANDO MUHAMMAD KHAN | 2014 | SINDH | 0 | 0.027806 | 3.715927 |
| THARPARKAR | 2014 | SINDH | 55.55556 | 42.72493 | 73.37234 |
| THATTA | 2014 | SINDH | 0 | 0.020846 | 3.494592 |
| TOBA TEK SINGH | 2014 | PUNJAB | 0 | 0.01629 | 3.753552 |
| UMER KOT | 2014 | SINDH | 6.622517 | 3.237345 | 13.58915 |
| VEHARI | 2014 | PUNJAB | 0 | 0.019845 | 3.693127 |
| ABBOTTABAD | 2015 | KPK | 3.822265 | 1.370682 | 10.16294 |
| ATTOCK | 2015 | PUNJAB | 0 | 0.026166 | 3.485943 |
| BADIN | 2015 | SINDH | 0 | 0.0213 | 3.894306 |
| BAHAWALNAGAR | 2015 | PUNJAB | 0.044352 | 0.023251 | 3.837315 |
| BAHAWALPUR | 2015 | PUNJAB | 0 | 0.026471 | 3.894597 |
| CHAKWAL | 2015 | PUNJAB | 0 | 0.030829 | 3.834359 |
| CHINIOT | 2015 | PUNJAB | 1.686341 | 0.540962 | 6.780273 |
| DADU | 2015 | SINDH | 3.72093 | 1.395236 | 10.23255 |
| DERA GHAZI KHAN | 2015 | PUNJAB | 1.048768 | 0.27885 | 5.583361 |
| D. I. KHAN | 2015 | KPK | 0 | 0.025502 | 3.888488 |
| FAISALABAD | 2015 | PUNJAB | 0.184248 | 0.043219 | 3.825863 |
| GHOTKI | 2015 | SINDH | 11.74743 | 6.460193 | 20.44328 |
| GUJRANWALA | 2015 | PUNJAB | 0.052754 | 0.021348 | 3.754619 |
| GUJRAT | 2015 | PUNJAB | 0.047621 | 0.029441 | 3.806562 |
| HAFIZABAD | 2015 | PUNJAB | 1.285347 | 0.314517 | 6.061108 |
| HARIPUR | 2015 | KPK | 1.569859 | 0.40877 | 6.137986 |
| HYDERABAD | 2015 | SINDH | 4.658761 | 2.167686 | 10.88409 |
| JACOBABAD | 2015 | SINDH | 0 | 0.030289 | 3.47261 |
| JHELUM | 2015 | PUNJAB | 0 | 0.022059 | 4.162176 |
| KAMBAR SHAHDAD KOT | 2015 | SINDH | 0 | 0.016989 | 3.510855 |
| CENTRAL KARACHI | 2015 | SINDH | 17.07696 | 10.93836 | 26.51916 |
| EAST KARACHI | 2015 | SINDH | 44.22315 | 32.03747 | 58.70696 |
| SOUTH KARACHI | 2015 | SINDH | 70.31308 | 56.61008 | 87.68574 |
| WEST KARACHI | 2015 | SINDH | 3.492031 | 1.289504 | 9.07519 |
| KASHMORE | 2015 | SINDH | 0 | 0.022983 | 3.853916 |
| KECH | 2015 | BALOCHISTAN | 8.62069 | 4.732143 | 16.20646 |
| KHAIRPUR | 2015 | SINDH | 4.716981 | 2.241333 | 11.31145 |
| KHANEWAL | 2015 | PUNJAB | 0 | 0.013758 | 3.695097 |
| KORANGI KARACHI | 2015 | SINDH | 9.195283 | 4.850219 | 16.92613 |
| LAHORE | 2015 | PUNJAB | 0.938222 | 0.225223 | 5.376834 |
| LARKANA | 2015 | SINDH | 5.009107 | 2.235055 | 12.17434 |
| LASBELA | 2015 | BALOCHISTAN | 83.96947 | 68.25521 | 102.6489 |
| LEIAH | 2015 | PUNJAB | 0.911577 | 0.210394 | 5.423475 |
| MALIR KARACHI | 2015 | SINDH | 5.234861 | 2.37381 | 11.94016 |
| MANDI BAHAUDDIN | 2015 | PUNJAB | 0 | 0.031475 | 3.695993 |
| MANSEHRA | 2015 | KPK | 5.714286 | 2.510572 | 12.64887 |
| MARDAN | 2015 | KPK | 0 | 0.025218 | 3.543388 |
| MATIARI | 2015 | SINDH | 0 | 0.016621 | 3.721838 |
| MIANWALI | 2015 | PUNJAB | 0 | 0.030468 | 3.469947 |
| MIRPUR KHAS | 2015 | SINDH | 5.36193 | 2.494687 | 11.81891 |
| MULTAN | 2015 | PUNJAB | 1.39579 | 0.359584 | 6.022283 |
| MUZAFFARGARH | 2015 | PUNJAB | 0.030769 | 0.037452 | 3.643667 |
| NANKANA SAHIB | 2015 | PUNJAB | 0 | 0.02697 | 3.518421 |
| NAUSHAHRO FEROZE | 2015 | SINDH | 1.208459 | 0.337329 | 5.869335 |
| NOWSHERA | 2015 | KPK | 1.312336 | 0.360703 | 6.058507 |
| OKARA | 2015 | PUNJAB | 1.229256 | 0.33872 | 5.595157 |
| PAKPATTAN | 2015 | PUNJAB | 0 | 0.020246 | 4.023844 |
| PESHAWAR | 2015 | KPK | 1.927365 | 0.661975 | 7.131465 |
| QUETTA | 2015 | BALOCHISTAN | 2.768166 | 0.995204 | 8.310981 |
| RAHIM YAR KHAN | 2015 | PUNJAB | 0.110272 | 0.03498 | 4.059296 |
| RAJANPUR | 2015 | PUNJAB | 0 | 0.02369 | 3.528509 |
| RAWALPINDI | 2015 | PUNJAB | 0.123083 | 0.047383 | 3.960865 |
| SAHIWAL | 2015 | PUNJAB | 0.209139 | 0.031933 | 4.374215 |
| SANGHAR | 2015 | SINDH | 1.017294 | 0.22678 | 5.159783 |
| SARGODHA | 2015 | PUNJAB | 0.035595 | 0.025173 | 3.85993 |
| SHAHEED BENAZIR ABAD | 2015 | SINDH | 0.247361 | 0.047387 | 4.248954 |
| SHEIKHUPURA | 2015 | PUNJAB | 0 | 0.026035 | 3.522173 |
| SHIKARPUR | 2015 | SINDH | 0.923361 | 0.230001 | 4.877783 |
| SIALKOT | 2015 | PUNJAB | 0 | 0.022346 | 3.303081 |
| SUJAWAL | 2015 | SINDH | 0 | 0.028697 | 3.635747 |
| SUKKUR | 2015 | SINDH | 6.063433 | 2.79295 | 13.05419 |
| SWABI | 2015 | KPK | 0 | 0.028168 | 3.309895 |
| TANDO ALLAHYAR | 2015 | SINDH | 0 | 0.023881 | 3.747945 |
| TANDO MUHAMMAD KHAN | 2015 | SINDH | 0 | 0.023546 | 3.979598 |
| THARPARKAR | 2015 | SINDH | 0 | 0.024348 | 3.749489 |
| THATTA | 2015 | SINDH | 2.55102 | 0.901358 | 8.230676 |
| TOBA TEK SINGH | 2015 | PUNJAB | 0.119825 | 0.039156 | 4.160447 |
| UMER KOT | 2015 | SINDH | 6.472492 | 3.328551 | 13.49024 |
| VEHARI | 2015 | PUNJAB | 0 | 0.033331 | 3.750145 |
| ABBOTTABAD | 2016 | KPK | 2.345216 | 0.735112 | 7.850927 |
| ATTOCK | 2016 | PUNJAB | 2.352941 | 0.864376 | 7.954756 |
| BADIN | 2016 | SINDH | 4.62963 | 2.013662 | 10.76589 |
| BAHAWALNAGAR | 2016 | PUNJAB | 0.043622 | 0.034371 | 3.676651 |
| BAHAWALPUR | 2016 | PUNJAB | 0.1063 | 0.035853 | 3.66982 |
| BANNU | 2016 | KPK | 0 | 0.023287 | 3.643484 |
| CHAKWAL | 2016 | PUNJAB | 0 | 0.025387 | 3.950098 |
| CHINIOT | 2016 | PUNJAB | 0 | 0.026424 | 4.20011 |
| DADU | 2016 | SINDH | 5.499542 | 2.283012 | 12.48122 |
| DERA GHAZI KHAN | 2016 | PUNJAB | 1.02459 | 0.221987 | 5.256985 |
| D. I. KHAN | 2016 | KPK | 0 | 0.020551 | 4.076557 |
| FAISALABAD | 2016 | PUNJAB | 0.214122 | 0.04661 | 4.274712 |
| GHOTKI | 2016 | SINDH | 17.24138 | 11.17328 | 27.46996 |
| GUJRANWALA | 2016 | PUNJAB | 0.025923 | 0.029867 | 3.630613 |
| GUJRAT | 2016 | PUNJAB | 0.140905 | 0.044699 | 3.738815 |
| HAFIZABAD | 2016 | PUNJAB | 2.531646 | 0.908831 | 7.695442 |
| HYDERABAD | 2016 | SINDH | 9.692573 | 5.344339 | 18.62298 |
| JACOBABAD | 2016 | SINDH | 4.754358 | 2.169857 | 10.58573 |
| JHANG | 2016 | PUNJAB | 2.358491 | 0.75573 | 7.552566 |
| JHELUM | 2016 | PUNJAB | 0 | 0.013035 | 3.727698 |
| KAMBAR SHAHDAD KOT | 2016 | SINDH | 0 | 0.028025 | 3.575896 |
| CENTRAL KARACHI | 2016 | SINDH | 20.63527 | 13.89779 | 31.62672 |
| EAST KARACHI | 2016 | SINDH | 47.85744 | 36.79151 | 61.64613 |
| SOUTH KARACHI | 2016 | SINDH | 56.25642 | 42.88834 | 71.00385 |
| WEST KARACHI | 2016 | SINDH | 2.967859 | 1.035211 | 9.126069 |
| KASHMORE | 2016 | SINDH | 0 | 0.025829 | 3.781106 |
| KECH | 2016 | BALOCHISTAN | 8.333333 | 4.432524 | 15.54817 |
| KHAIRPUR | 2016 | SINDH | 3.304693 | 1.2534 | 9.328614 |
| KHANEWAL | 2016 | PUNJAB | 0 | 0.032375 | 4.034497 |
| KORANGI KARACHI | 2016 | SINDH | 13.38763 | 8.231282 | 23.02591 |
| LAHORE | 2016 | PUNJAB | 13.60656 | 8.173244 | 22.22255 |
| LARKANA | 2016 | SINDH | 5.811354 | 2.558709 | 12.53179 |
| LASBELA | 2016 | BALOCHISTAN | 268.6567 | 242.1288 | 295.7427 |
| LEIAH | 2016 | PUNJAB | 0 | 0.023488 | 3.766953 |
| MALAKAND | 2016 | KPK | 0.21645 | 0.045932 | 4.274341 |
| MALIR KARACHI | 2016 | SINDH | 10.17182 | 5.392802 | 18.22468 |
| MANDI BAHAUDDIN | 2016 | PUNJAB | 0 | 0.011304 | 3.917726 |
| MANSEHRA | 2016 | KPK | 2.80112 | 0.957002 | 8.328454 |
| MARDAN | 2016 | KPK | 1.321004 | 0.385137 | 6.599056 |
| MATIARI | 2016 | SINDH | 0 | 0.034247 | 3.506838 |
| MIANWALI | 2016 | PUNJAB | 0 | 0.026057 | 3.776242 |
| MIRPUR KHAS | 2016 | SINDH | 6.145742 | 3.048367 | 12.82787 |
| MULTAN | 2016 | PUNJAB | 0.164411 | 0.036066 | 4.115739 |
| MUZAFFARGARH | 2016 | PUNJAB | 0.030123 | 0.024928 | 3.67222 |
| NANKANA SAHIB | 2016 | PUNJAB | 2.331002 | 0.746423 | 7.832731 |
| NAUSHAHRO FEROZE | 2016 | SINDH | 1.781473 | 0.505854 | 7.330742 |
| NOWSHERA | 2016 | KPK | 0 | 0.033768 | 3.708995 |
| OKARA | 2016 | PUNJAB | 1.212121 | 0.299394 | 6.01768 |
| PAKPATTAN | 2016 | PUNJAB | 1.27551 | 0.319166 | 6.118891 |
| PESHAWAR | 2016 | KPK | 1.211676 | 0.306743 | 6.072644 |
| QUETTA | 2016 | BALOCHISTAN | 6.671114 | 3.117819 | 14.0577 |
| RAHIM YAR KHAN | 2016 | PUNJAB | 0 | 0.029101 | 3.797661 |
| RAWALPINDI | 2016 | PUNJAB | 0.120589 | 0.032054 | 3.785094 |
| SAHIWAL | 2016 | PUNJAB | 0.206164 | 0.059218 | 3.869755 |
| SANGHAR | 2016 | SINDH | 2.992519 | 0.999767 | 8.971489 |
| SARGODHA | 2016 | PUNJAB | 0.070129 | 0.025123 | 3.826275 |
| SHAHEED BENAZIR ABAD | 2016 | SINDH | 0.243171 | 0.077406 | 3.820613 |
| SHEIKHUPURA | 2016 | PUNJAB | 0.187928 | 0.04367 | 4.16924 |
| SHIKARPUR | 2016 | SINDH | 4.545455 | 1.85541 | 10.99908 |
| SIALKOT | 2016 | PUNJAB | 0 | 0.021304 | 3.544444 |
| SUJAWAL | 2016 | SINDH | 6.896552 | 3.43205 | 14.01613 |
| SUKKUR | 2016 | SINDH | 11.883 | 6.433862 | 20.56266 |
| SWABI | 2016 | KPK | 0 | 0.027677 | 3.565646 |
| SWAT | 2016 | KPK | 0 | 0.022075 | 4.007579 |
| TANDO ALLAHYAR | 2016 | SINDH | 1.449275 | 0.452478 | 6.206678 |
| TANDO MUHAMMAD KHAN | 2016 | SINDH | 3.003003 | 1.04001 | 8.529082 |
| THARPARKAR | 2016 | SINDH | 315.7895 | 286.1325 | 345.2864 |
| THATTA | 2016 | SINDH | 0 | 0.027901 | 3.541543 |
| TOBA TEK SINGH | 2016 | PUNJAB | 0.177242 | 0.031722 | 3.832251 |
| UMER KOT | 2016 | SINDH | 19.04762 | 12.23155 | 28.89446 |
| ABBOTTABAD | 2017 | KPK | 0.921234 | 0.191774 | 5.159315 |
| ATTOCK | 2017 | PUNJAB | 2.314815 | 0.692421 | 7.84879 |
| BADIN | 2017 | SINDH | 4.535147 | 1.752204 | 10.72345 |
| BAHAWALNAGAR | 2017 | PUNJAB | 0 | 0.031418 | 3.751604 |
| BAHAWALPUR | 2017 | PUNJAB | 0.034804 | 0.027812 | 3.817975 |
| BANNU | 2017 | KPK | 2.145923 | 0.584871 | 7.701801 |
| BUNER | 2017 | KPK | 0 | 0.027001 | 3.344666 |
| CHAKWAL | 2017 | PUNJAB | 0 | 0.013363 | 3.775345 |
| CHINIOT | 2017 | PUNJAB | 0 | 0.019677 | 3.889703 |
| DADU | 2017 | SINDH | 0.902527 | 0.168979 | 5.480023 |
| DERA GHAZI KHAN | 2017 | PUNJAB | 1.001502 | 0.203431 | 5.884418 |
| D. I. KHAN | 2017 | KPK | 0 | 0.024737 | 3.874026 |
| FAISALABAD | 2017 | PUNJAB | 0.178213 | 0.046208 | 4.058989 |
| GHOTKI | 2017 | SINDH | 16.85393 | 10.84052 | 26.96875 |
| GUJRANWALA | 2017 | PUNJAB | 0.152909 | 0.035629 | 3.645025 |
| GUJRAT | 2017 | PUNJAB | 0.046333 | 0.040834 | 3.414282 |
| HAFIZABAD | 2017 | PUNJAB | 4.987531 | 2.260558 | 11.72242 |
| HARIPUR | 2017 | KPK | 0 | 0.025776 | 3.548737 |
| HYDERABAD | 2017 | SINDH | 4.735457 | 1.993412 | 10.95523 |
| JACOBABAD | 2017 | SINDH | 1.5625 | 0.424539 | 6.622719 |
| JHANG | 2017 | PUNJAB | 1.15942 | 0.327756 | 5.562303 |
| JHELUM | 2017 | PUNJAB | 0 | 0.028347 | 3.782987 |
| KAMBAR SHAHDAD KOT | 2017 | SINDH | 0 | 0.031107 | 3.87506 |
| CENTRAL KARACHI | 2017 | SINDH | 12.55194 | 7.480007 | 21.7656 |
| EAST KARACHI | 2017 | SINDH | 20.35157 | 13.17226 | 31.32691 |
| SOUTH KARACHI | 2017 | SINDH | 32.49255 | 22.63843 | 45.2307 |
| WEST KARACHI | 2017 | SINDH | 0.888772 | 0.20734 | 5.236002 |
| KASHMORE | 2017 | SINDH | 8.02139 | 4.35309 | 15.34454 |
| KHAIRPUR | 2017 | SINDH | 2.594034 | 0.819095 | 8.528718 |
| KHANEWAL | 2017 | PUNJAB | 0.639386 | 0.137848 | 4.662752 |
| KOHAT | 2017 | KPK | 0 | 0.024419 | 3.628657 |
| KORANGI KARACHI | 2017 | SINDH | 2.045704 | 0.627936 | 6.62771 |
| LAHORE | 2017 | PUNJAB | 1.102498 | 0.270745 | 5.697322 |
| LARKANA | 2017 | SINDH | 7.901668 | 3.969852 | 15.36172 |
| LASBELA | 2017 | BALOCHISTAN | 29.19708 | 20.67254 | 41.94156 |
| LEIAH | 2017 | PUNJAB | 1.748252 | 0.505362 | 6.498809 |
| LOWER DIR | 2017 | KPK | 0 | 0.025872 | 3.70717 |
| MALIR KARACHI | 2017 | SINDH | 3.07425 | 1.097788 | 9.013106 |
| MANSEHRA | 2017 | KPK | 2.747253 | 0.93065 | 8.464074 |
| MARDAN | 2017 | KPK | 0 | 0.021141 | 3.675594 |
| MATIARI | 2017 | SINDH | 0 | 0.017877 | 3.300341 |
| MIANWALI | 2017 | PUNJAB | 0 | 0.024725 | 3.889656 |
| MIRPUR KHAS | 2017 | SINDH | 1.725626 | 0.487225 | 6.656569 |
| MULTAN | 2017 | PUNJAB | 0.161442 | 0.059224 | 3.945442 |
| MUZAFFARGARH | 2017 | PUNJAB | 0.029503 | 0.021933 | 3.60735 |
| NANKANA SAHIB | 2017 | PUNJAB | 0 | 0.019218 | 3.717538 |
| NAUSHAHRO FEROZE | 2017 | SINDH | 1.751313 | 0.536058 | 6.355425 |
| NOWSHERA | 2017 | KPK | 1.253133 | 0.359987 | 6.08751 |
| OKARA | 2017 | PUNJAB | 1.194743 | 0.28398 | 6.034725 |
| PAKPATTAN | 2017 | PUNJAB | 0 | 0.030687 | 3.808251 |
| PESHAWAR | 2017 | KPK | 3.534578 | 1.365134 | 8.919677 |
| QUETTA | 2017 | BALOCHISTAN | 6.443299 | 2.871116 | 13.50927 |
| RAHIM YAR KHAN | 2017 | PUNJAB | 0.132813 | 0.039115 | 3.739231 |
| RAJANPUR | 2017 | PUNJAB | 1.228501 | 0.324086 | 5.865142 |
| RAWALPINDI | 2017 | PUNJAB | 0.023638 | 0.044429 | 3.837477 |
| SAHIWAL | 2017 | PUNJAB | 0.050818 | 0.024776 | 3.771478 |
| SANGHAR | 2017 | SINDH | 0.489476 | 0.080026 | 4.465067 |
| SARGODHA | 2017 | PUNJAB | 0.103652 | 0.034245 | 3.566221 |
| SHAHEED BENAZIR ABAD | 2017 | SINDH | 0.47824 | 0.100529 | 4.421994 |
| SHEIKHUPURA | 2017 | PUNJAB | 0.295268 | 0.070964 | 3.887123 |
| SHIKARPUR | 2017 | SINDH | 0 | 0.025969 | 3.996367 |
| SIALKOT | 2017 | PUNJAB | 0.098364 | 0.041502 | 3.550585 |
| SUJAWAL | 2017 | SINDH | 0 | 0.041584 | 3.822203 |
| SUKKUR | 2017 | SINDH | 5.824373 | 2.642823 | 12.7931 |
| SWABI | 2017 | KPK | 0 | 0.020251 | 3.546523 |
| TANDO ALLAHYAR | 2017 | SINDH | 7.092199 | 3.394054 | 14.91461 |
| TANDO MUHAMMAD KHAN | 2017 | SINDH | 2.949853 | 0.919004 | 8.4757 |
| THARPARKAR | 2017 | SINDH | 157.8947 | 136.9189 | 181.8423 |
| THATTA | 2017 | SINDH | 2.444988 | 0.761621 | 7.697467 |
| TOBA TEK SINGH | 2017 | PUNJAB | 0.116543 | 0.032617 | 3.878519 |
| UMER KOT | 2017 | SINDH | 3.10559 | 1.074123 | 8.548779 |
| VEHARI | 2017 | PUNJAB | 0.597372 | 0.123461 | 4.541175 |
| ABBOTTABAD | 2018 | KPK | 0.431406 | 0.092167 | 4.46345 |
| ATTOCK | 2018 | PUNJAB | 3.992016 | 1.427255 | 10.45352 |
| BADIN | 2018 | SINDH | 0 | 0.021983 | 3.732341 |
| BAHAWALNAGAR | 2018 | PUNJAB | 0.071332 | 0.034446 | 4.228815 |
| BAHAWALPUR | 2018 | PUNJAB | 0 | 0.035165 | 3.468737 |
| BANNU | 2018 | KPK | 0 | 0.033564 | 3.558517 |
| BHAKKAR | 2018 | PUNJAB | 0 | 0.030451 | 3.700097 |
| BUNER | 2018 | KPK | 0 | 0.025086 | 3.399557 |
| CHAKWAL | 2018 | PUNJAB | 2.12766 | 0.620574 | 6.998143 |
| DADU | 2018 | SINDH | 3.192338 | 1.124057 | 8.772771 |
| DERA GHAZI KHAN | 2018 | PUNJAB | 0.420521 | 0.094823 | 4.378624 |
| D. I. KHAN | 2018 | KPK | 0 | 0.02361 | 3.552998 |
| FAISALABAD | 2018 | PUNJAB | 0.09782 | 0.028613 | 3.887559 |
| GHOTKI | 2018 | SINDH | 20.83333 | 13.55242 | 30.67587 |
| GUJRANWALA | 2018 | PUNJAB | 0.0425 | 0.032127 | 3.687937 |
| GUJRAT | 2018 | PUNJAB | 0.03937 | 0.029592 | 3.70772 |
| HAFIZABAD | 2018 | PUNJAB | 0 | 0.01857 | 3.598305 |
| HARIPUR | 2018 | KPK | 0 | 0.023341 | 3.508109 |
| HYDERABAD | 2018 | SINDH | 2.52512 | 0.854683 | 7.584755 |
| JACOBABAD | 2018 | SINDH | 3.989362 | 1.48665 | 9.968382 |
| JHANG | 2018 | PUNJAB | 0 | 0.021601 | 3.886823 |
| JHELUM | 2018 | PUNJAB | 0 | 0.025771 | 3.364796 |
| CENTRAL KARACHI | 2018 | SINDH | 6.917423 | 3.344154 | 14.24186 |
| EAST KARACHI | 2018 | SINDH | 9.886688 | 5.427458 | 18.0201 |
| SOUTH KARACHI | 2018 | SINDH | 16.73572 | 10.67771 | 26.78182 |
| WEST KARACHI | 2018 | SINDH | 1.328273 | 0.312541 | 6.262135 |
| KASHMORE | 2018 | SINDH | 5.868545 | 2.674256 | 12.42858 |
| KECH | 2018 | BALOCHISTAN | 20.68966 | 13.51363 | 31.53183 |
| KHAIRPUR | 2018 | SINDH | 3.588517 | 1.478537 | 9.495345 |
| KHANEWAL | 2018 | PUNJAB | 0 | 0.020745 | 3.95689 |
| KHUSHAB | 2018 | PUNJAB | 0 | 0.034173 | 3.461848 |
| KORANGI KARACHI | 2018 | SINDH | 0.773773 | 0.142414 | 5.251176 |
| LAHORE | 2018 | PUNJAB | 0.428825 | 0.105649 | 4.535155 |
| LARKANA | 2018 | SINDH | 7.060572 | 3.400829 | 14.52636 |
| LASBELA | 2018 | BALOCHISTAN | 18.40491 | 11.65191 | 29.00096 |
| LEIAH | 2018 | PUNJAB | 0 | 0.025895 | 3.858947 |
| LOWER DIR | 2018 | KPK | 5.050505 | 2.108076 | 11.67923 |
| MALIR KARACHI | 2018 | SINDH | 1.376535 | 0.358561 | 5.984145 |
| MANDI BAHAUDDIN | 2018 | PUNJAB | 0 | 0.026977 | 3.864686 |
| MANSEHRA | 2018 | KPK | 2.358491 | 0.668203 | 8.096164 |
| MARDAN | 2018 | KPK | 0 | 0.030955 | 3.859841 |
| MATIARI | 2018 | SINDH | 4.938272 | 2.178937 | 11.40672 |
| MIANWALI | 2018 | PUNJAB | 3.099174 | 1.24297 | 8.662893 |
| MIRPUR KHAS | 2018 | SINDH | 1.536098 | 0.465362 | 6.341406 |
| MULTAN | 2018 | PUNJAB | 0.071016 | 0.037012 | 3.487384 |
| MUZAFFARGARH | 2018 | PUNJAB | 0 | 0.029463 | 3.569899 |
| NANKANA SAHIB | 2018 | PUNJAB | 0 | 0.025417 | 3.830678 |
| NAUSHAHRO FEROZE | 2018 | SINDH | 1.580611 | 0.479192 | 6.216529 |
| NOWSHERA | 2018 | KPK | 0 | 0.033549 | 3.524119 |
| OKARA | 2018 | PUNJAB | 0.513084 | 0.148677 | 4.663018 |
| PAKPATTAN | 2018 | PUNJAB | 1.064963 | 0.219642 | 5.487078 |
| PESHAWAR | 2018 | KPK | 0.254915 | 0.059725 | 3.9959 |
| QUETTA | 2018 | BALOCHISTAN | 5.036374 | 2.187485 | 11.48971 |
| RAHIM YAR KHAN | 2018 | PUNJAB | 0 | 0.019723 | 3.949473 |
| RAJANPUR | 2018 | PUNJAB | 0 | 0.021715 | 3.274341 |
| RAWALPINDI | 2018 | PUNJAB | 0.08297 | 0.035951 | 3.661928 |
| SAHIWAL | 2018 | PUNJAB | 0 | 0.020557 | 3.659074 |
| SANGHAR | 2018 | SINDH | 0 | 0.023307 | 3.699745 |
| SARGODHA | 2018 | PUNJAB | 0 | 0.024785 | 3.421273 |
| SHAHEED BENAZIR ABAD | 2018 | SINDH | 0.484731 | 0.084701 | 4.540051 |
| SHEIKHUPURA | 2018 | PUNJAB | 0.031297 | 0.029032 | 4.183562 |
| SHIKARPUR | 2018 | SINDH | 2.377179 | 0.751517 | 7.35627 |
| SIALKOT | 2018 | PUNJAB | 0.028183 | 0.026167 | 3.678922 |
| SUJAWAL | 2018 | SINDH | 6.289308 | 2.873684 | 13.54557 |
| SUKKUR | 2018 | SINDH | 5.261028 | 2.404148 | 12.03124 |
| TANDO ALLAHYAR | 2018 | SINDH | 1.283697 | 0.324509 | 5.841207 |
| TANDO MUHAMMAD KHAN | 2018 | SINDH | 0 | 0.027975 | 3.537312 |
| THARPARKAR | 2018 | SINDH | 0 | 0.021794 | 4.2467 |
| THATTA | 2018 | SINDH | 0 | 0.028414 | 3.569649 |
| TOBA TEK SINGH | 2018 | PUNJAB | 0.0505 | 0.030234 | 4.100976 |
| UMER KOT | 2018 | SINDH | 0 | 0.037452 | 3.815698 |
| ABBOTTABAD | 2019 | KPK | 0.860215 | 0.217151 | 5.06831 |
| ATTOCK | 2019 | PUNJAB | 1.984127 | 0.621359 | 7.408192 |
| BADIN | 2019 | SINDH | 8.350731 | 4.531313 | 16.46824 |
| BAHAWALNAGAR | 2019 | PUNJAB | 0.176835 | 0.042816 | 3.584336 |
| BAHAWALPUR | 2019 | PUNJAB | 0.117436 | 0.043928 | 4.036163 |
| BANNU | 2019 | KPK | 0.911577 | 0.186662 | 4.873991 |
| BHAKKAR | 2019 | PUNJAB | 0 | 0.021633 | 3.667447 |
| BUNER | 2019 | KPK | 0 | 0.025705 | 3.55767 |
| CHAKWAL | 2019 | PUNJAB | 2.114165 | 0.759542 | 7.298334 |
| CHARSADDA | 2019 | KPK | 0 | 0.029513 | 3.544892 |
| CHINIOT | 2019 | PUNJAB | 0 | 0.023372 | 3.622988 |
| DADU | 2019 | SINDH | 14.28571 | 8.428735 | 24.12879 |
| DERA GHAZI KHAN | 2019 | PUNJAB | 2.085941 | 0.666014 | 7.127643 |
| D. I. KHAN | 2019 | KPK | 1.503759 | 0.429951 | 5.835523 |
| FAISALABAD | 2019 | PUNJAB | 0.222062 | 0.062068 | 4.308594 |
| GHOTKI | 2019 | SINDH | 38.91051 | 28.15954 | 51.39263 |
| GUJRANWALA | 2019 | PUNJAB | 0.295047 | 0.048389 | 4.363951 |
| GUJRAT | 2019 | PUNJAB | 0.273534 | 0.06752 | 4.190272 |
| HAFIZABAD | 2019 | PUNJAB | 2.159827 | 0.73847 | 7.382102 |
| HARIPUR | 2019 | KPK | 0 | 0.021 | 3.654324 |
| HYDERABAD | 2019 | SINDH | 16.64573 | 10.18096 | 26.29115 |
| JACOBABAD | 2019 | SINDH | 13.19261 | 8.056125 | 22.40119 |
| JAMSHORO | 2019 | SINDH | 4.694836 | 1.893047 | 11.05276 |
| JHANG | 2019 | PUNJAB | 4.085802 | 1.719366 | 9.928725 |
| JHELUM | 2019 | PUNJAB | 1.036269 | 0.263714 | 5.255559 |
| KAMBAR SHAHDAD KOT | 2019 | SINDH | 2.386635 | 0.751978 | 7.842979 |
| CENTRAL KARACHI | 2019 | SINDH | 82.38814 | 66.584 | 102.1205 |
| EAST KARACHI | 2019 | SINDH | 117.4944 | 98.05691 | 137.7304 |
| SOUTH KARACHI | 2019 | SINDH | 135.0813 | 114.6549 | 157.7955 |
| WEST KARACHI | 2019 | SINDH | 10.50361 | 5.712442 | 18.48405 |
| KASHMORE | 2019 | SINDH | 19.83664 | 12.4444 | 30.52072 |
| KASUR | 2019 | PUNJAB | 1.01833 | 0.284808 | 5.585406 |
| KECH | 2019 | BALOCHISTAN | 54.79452 | 41.31339 | 71.08904 |
| KHAIRPUR | 2019 | SINDH | 20.85816 | 13.61136 | 33.30794 |
| KHANEWAL | 2019 | PUNJAB | 1.094691 | 0.33888 | 5.925504 |
| KHUSHAB | 2019 | PUNJAB | 0 | 0.02102 | 3.753171 |
| KORANGI KARACHI | 2019 | SINDH | 36.64097 | 26.81368 | 50.77308 |
| LAHORE | 2019 | PUNJAB | 2.640692 | 0.795875 | 7.89688 |
| LARKANA | 2019 | SINDH | 34.29204 | 24.90394 | 46.75383 |
| LASBELA | 2019 | BALOCHISTAN | 175.7576 | 154.2414 | 201.3505 |
| LEIAH | 2019 | PUNJAB | 2.243829 | 0.740669 | 7.575575 |
| LODHRAN | 2019 | PUNJAB | 1.295337 | 0.289397 | 5.91911 |
| LORALAI | 2019 | BALOCHISTAN | 0 | 0.013791 | 3.813932 |
| LOWER DIR | 2019 | KPK | 5.025126 | 2.396183 | 11.6564 |
| MALAKAND | 2019 | KPK | 0 | 0.021263 | 3.422227 |
| MALIR KARACHI | 2019 | SINDH | 37.46594 | 27.2636 | 52.55294 |
| MANDI BAHAUDDIN | 2019 | PUNJAB | 9.969325 | 5.905618 | 17.70446 |
| MANSEHRA | 2019 | KPK | 2.34192 | 0.739656 | 7.920436 |
| MARDAN | 2019 | KPK | 1.103347 | 0.28524 | 5.541728 |
| MATIARI | 2019 | SINDH | 12.28501 | 6.653888 | 20.73519 |
| MIANWALI | 2019 | PUNJAB | 5.122951 | 2.254893 | 12.08376 |
| MIRPUR KHAS | 2019 | SINDH | 12.21374 | 6.875868 | 21.14261 |
| MULTAN | 2019 | PUNJAB | 0.47061 | 0.083488 | 4.589524 |
| MUZAFFARGARH | 2019 | PUNJAB | 0.050202 | 0.035204 | 3.854619 |
| NANKANA SAHIB | 2019 | PUNJAB | 0 | 0.02639 | 3.53329 |
| NAUSHAHRO FEROZE | 2019 | SINDH | 8.914525 | 4.602674 | 16.88656 |
| NOWSHERA | 2019 | KPK | 22.67819 | 15.04381 | 34.54675 |
| OKARA | 2019 | PUNJAB | 2.54842 | 0.86463 | 8.194653 |
| PAKPATTAN | 2019 | PUNJAB | 3.171247 | 1.146474 | 8.926422 |
| PESHAWAR | 2019 | KPK | 5.296543 | 2.389837 | 12.14956 |
| QUETTA | 2019 | BALOCHISTAN | 26.68149 | 18.4809 | 37.92352 |
| RAHIM YAR KHAN | 2019 | PUNJAB | 0.361999 | 0.058106 | 4.331339 |
| RAJANPUR | 2019 | PUNJAB | 1.012146 | 0.232082 | 5.682291 |
| RAWALPINDI | 2019 | PUNJAB | 0.659726 | 0.145552 | 5.320743 |
| SAHIWAL | 2019 | PUNJAB | 0.218627 | 0.046922 | 3.752539 |
| SANGHAR | 2019 | SINDH | 3.436426 | 1.259538 | 9.684229 |
| SARGODHA | 2019 | PUNJAB | 0.116053 | 0.038072 | 3.908223 |
| SHAHEED BENAZIR ABAD | 2019 | SINDH | 1.926252 | 0.498641 | 7.212751 |
| SHEIKHUPURA | 2019 | PUNJAB | 0.279547 | 0.072624 | 4.283867 |
| SHIKARPUR | 2019 | SINDH | 7.874016 | 4.079197 | 15.05435 |
| SIALKOT | 2019 | PUNJAB | 0.055974 | 0.032234 | 3.853529 |
| SUJAWAL | 2019 | SINDH | 12.5 | 7.414639 | 21.59353 |
| SUKKUR | 2019 | SINDH | 30.2054 | 22.02435 | 42.74915 |
| SWABI | 2019 | KPK | 46.51163 | 34.37946 | 62.54834 |
| SWAT | 2019 | KPK | 0 | 0.022966 | 3.800949 |
| TANDO ALLAHYAR | 2019 | SINDH | 6.385696 | 3.115161 | 13.95329 |
| TANDO MUHAMMAD KHAN | 2019 | SINDH | 5.464481 | 2.455671 | 12.30572 |
| THARPARKAR | 2019 | SINDH | 47.61905 | 36.1833 | 62.13125 |
| THATTA | 2019 | SINDH | 32.89474 | 23.20999 | 45.45635 |
| TOBA TEK SINGH | 2019 | PUNJAB | 0.050166 | 0.027594 | 3.649477 |
| UMER KOT | 2019 | SINDH | 11.42857 | 6.271029 | 20.77671 |
| VEHARI | 2019 | PUNJAB | 0.995025 | 0.263117 | 5.449414 |
| ABBOTTABAD | 2020 | KPK | 0 | 0.018487 | 4.155084 |
| ATTOCK | 2020 | PUNJAB | 0 | 0.021408 | 3.651457 |
| BADIN | 2020 | SINDH | 2.079002 | 0.671126 | 7.179519 |
| BAHAWALNAGAR | 2020 | PUNJAB | 0.035073 | 0.027891 | 3.91961 |
| BAHAWALPUR | 2020 | PUNJAB | 0.029142 | 0.035408 | 3.418401 |
| BHAKKAR | 2020 | PUNJAB | 0 | 0.033191 | 3.458628 |
| CHAKWAL | 2020 | PUNJAB | 0 | 0.029524 | 3.773171 |
| CHINIOT | 2020 | PUNJAB | 0.701754 | 0.149584 | 4.957456 |
| DADU | 2020 | SINDH | 0.789266 | 0.206461 | 5.041744 |
| DERA GHAZI KHAN | 2020 | PUNJAB | 0 | 0.025292 | 3.784008 |
| D. I. KHAN | 2020 | KPK | 0 | 0.018921 | 3.223138 |
| FAISALABAD | 2020 | PUNJAB | 0.013785 | 0.032476 | 3.684275 |
| GHOTKI | 2020 | SINDH | 10.33592 | 5.392865 | 18.12942 |
| GUJRANWALA | 2020 | PUNJAB | 0.062708 | 0.040316 | 3.779601 |
| GUJRAT | 2020 | PUNJAB | 0 | 0.019725 | 3.597478 |
| HAFIZABAD | 2020 | PUNJAB | 0 | 0.024978 | 3.871298 |
| HYDERABAD | 2020 | SINDH | 3.177083 | 1.235368 | 9.177959 |
| JACOBABAD | 2020 | SINDH | 3.931848 | 1.487144 | 9.963611 |
| JHANG | 2020 | PUNJAB | 0 | 0.029263 | 3.341423 |
| JHELUM | 2020 | PUNJAB | 0 | 0.021095 | 3.880139 |
| KAMBAR SHAHDAD KOT | 2020 | SINDH | 2.369668 | 0.791983 | 7.789982 |
| CENTRAL KARACHI | 2020 | SINDH | 13.39184 | 8.10617 | 22.41027 |
| EAST KARACHI | 2020 | SINDH | 33.71523 | 23.6944 | 45.87799 |
| SOUTH KARACHI | 2020 | SINDH | 43.92916 | 33.10548 | 56.84289 |
| WEST KARACHI | 2020 | SINDH | 1.807815 | 0.475895 | 6.655197 |
| KASHMORE | 2020 | SINDH | 3.480278 | 1.451887 | 9.606927 |
| KECH | 2020 | BALOCHISTAN | 0 | 0.029267 | 4.203223 |
| KHAIRPUR | 2020 | SINDH | 4.154303 | 1.713238 | 10.61418 |
| KHANEWAL | 2020 | PUNJAB | 0 | 0.020652 | 3.452382 |
| KHUSHAB | 2020 | PUNJAB | 0 | 0.031516 | 3.532177 |
| KOHAT | 2020 | KPK | 0 | 0.028074 | 3.719246 |
| KORANGI KARACHI | 2020 | SINDH | 4.438935 | 2.067436 | 10.45969 |
| LAHORE | 2020 | PUNJAB | 0.307223 | 0.063266 | 4.497139 |
| LARKANA | 2020 | SINDH | 4.756678 | 2.201513 | 11.62469 |
| LASBELA | 2020 | BALOCHISTAN | 0 | 0.024746 | 3.592266 |
| LEIAH | 2020 | PUNJAB | 0 | 0.025795 | 3.76112 |
| MALAKAND | 2020 | KPK | 0 | 0.031617 | 3.515652 |
| MALIR KARACHI | 2020 | SINDH | 4.46035 | 1.930725 | 11.00321 |
| MANDI BAHAUDDIN | 2020 | PUNJAB | 0 | 0.025962 | 3.6586 |
| MANSEHRA | 2020 | KPK | 2.325581 | 0.846128 | 7.846215 |
| MARDAN | 2020 | KPK | 0 | 0.022013 | 3.530817 |
| MATIARI | 2020 | SINDH | 4.889976 | 2.062204 | 11.14788 |
| MIANWALI | 2020 | PUNJAB | 1.017294 | 0.266973 | 5.546193 |
| MIRPUR KHAS | 2020 | SINDH | 6.833713 | 3.370161 | 15.00519 |
| MULTAN | 2020 | PUNJAB | 0.046781 | 0.028683 | 3.661454 |
| MUZAFFARGARH | 2020 | PUNJAB | 0 | 0.022118 | 3.827277 |
| NANKANA SAHIB | 2020 | PUNJAB | 0 | 0.021931 | 3.54956 |
| NAUSHAHRO FEROZE | 2020 | SINDH | 1.043297 | 0.198846 | 5.647346 |
| NOWSHERA | 2020 | KPK | 0 | 0.021954 | 3.205525 |
| OKARA | 2020 | PUNJAB | 0 | 0.020353 | 3.758335 |
| PAKPATTAN | 2020 | PUNJAB | 0 | 0.016821 | 3.306632 |
| PESHAWAR | 2020 | KPK | 0.284118 | 0.074771 | 4.235696 |
| QUETTA | 2020 | BALOCHISTAN | 3.865268 | 1.493548 | 9.948742 |
| RAHIM YAR KHAN | 2020 | PUNJAB | 0.022466 | 0.02801 | 3.889733 |
| RAJANPUR | 2020 | PUNJAB | 0 | 0.019863 | 3.833828 |
| RAWALPINDI | 2020 | PUNJAB | 0.061475 | 0.028012 | 3.787741 |
| SAHIWAL | 2020 | PUNJAB | 0 | 0.020397 | 3.759475 |
| SANGHAR | 2020 | SINDH | 0.427168 | 0.088869 | 4.362282 |
| SARGODHA | 2020 | PUNJAB | 0 | 0.028558 | 3.640832 |
| SHAHEED BENAZIR ABAD | 2020 | SINDH | 0.205044 | 0.054959 | 4.133293 |
| SHEIKHUPURA | 2020 | PUNJAB | 0 | 0.026355 | 3.533485 |
| SHIKARPUR | 2020 | SINDH | 3.132341 | 1.160934 | 9.165976 |
| SIALKOT | 2020 | PUNJAB | 0 | 0.025696 | 3.674532 |
| SUJAWAL | 2020 | SINDH | 6.21118 | 2.972015 | 13.09404 |
| SUKKUR | 2020 | SINDH | 7.214429 | 3.429614 | 14.25037 |
| SWABI | 2020 | KPK | 0 | 0.023454 | 3.707201 |
| TANDO ALLAHYAR | 2020 | SINDH | 1.272265 | 0.291244 | 6.342322 |
| TANDO MUHAMMAD KHAN | 2020 | SINDH | 0 | 0.039748 | 3.603348 |
| THARPARKAR | 2020 | SINDH | 190.4762 | 167.4179 | 213.3793 |
| THATTA | 2020 | SINDH | 2.183406 | 0.685826 | 7.677043 |
| TOBA TEK SINGH | 2020 | PUNJAB | 0.049836 | 0.028718 | 3.732285 |
| UMER KOT | 2020 | SINDH | 5.681818 | 2.55708 | 12.11915 |
| VEHARI | 2020 | PUNJAB | 0 | 0.023259 | 3.874942 |
| ABBOTTABAD | 2021 | KPK | 0.42735 | 0.098942 | 4.271518 |
| ATTOCK | 2021 | PUNJAB | 3.913894 | 1.566119 | 9.805924 |
| BADIN | 2021 | SINDH | 8.281573 | 4.25723 | 16.00915 |
| BAHAWALNAGAR | 2021 | PUNJAB | 0.104355 | 0.035142 | 3.830215 |
| BAHAWALPUR | 2021 | PUNJAB | 0.057855 | 0.037898 | 4.02857 |
| BAJAUR | 2021 | KPK | 4.56621 | 1.819445 | 11.16675 |
| BANNU | 2021 | KPK | 0 | 0.015741 | 3.6084 |
| BHAKKAR | 2021 | PUNJAB | 4.237288 | 1.818833 | 9.517832 |
| CHARSADDA | 2021 | KPK | 1.277139 | 0.332657 | 5.915424 |
| CHINIOT | 2021 | PUNJAB | 4.1841 | 1.65978 | 10.72231 |
| DADU | 2021 | SINDH | 9.419152 | 4.920506 | 17.25263 |
| DERA GHAZI KHAN | 2021 | PUNJAB | 6.157635 | 2.922595 | 13.00238 |
| D. I. KHAN | 2021 | KPK | 0.746269 | 0.181074 | 5.0721 |
| FAISALABAD | 2021 | PUNJAB | 0.616135 | 0.113111 | 4.927401 |
| GHOTKI | 2021 | SINDH | 43.75804 | 33.20625 | 57.91065 |
| GUJRANWALA | 2021 | PUNJAB | 2.695306 | 0.940887 | 8.065173 |
| GUJRAT | 2021 | PUNJAB | 0.539021 | 0.113395 | 4.719916 |
| HAFIZABAD | 2021 | PUNJAB | 10.66098 | 6.061072 | 18.94811 |
| HARIPUR | 2021 | KPK | 0 | 0.019213 | 3.255793 |
| HYDERABAD | 2021 | SINDH | 17.98393 | 11.22652 | 28.1939 |
| JACOBABAD | 2021 | SINDH | 11.70351 | 6.833094 | 20.2544 |
| JAFFARABAD | 2021 | BALOCHISTAN | 15 | 9.08391 | 24.18606 |
| JAMSHORO | 2021 | SINDH | 9.302326 | 4.867336 | 18.06355 |
| JHANG | 2021 | PUNJAB | 3.030303 | 1.007069 | 8.700955 |
| JHELUM | 2021 | PUNJAB | 0 | 0.02703 | 3.606212 |
| KAMBAR SHAHDAD KOT | 2021 | SINDH | 4.716981 | 2.132784 | 11.86321 |
| CENTRAL KARACHI | 2021 | SINDH | 29.82676 | 21.47106 | 42.51256 |
| EAST KARACHI | 2021 | SINDH | 48.31706 | 37.04663 | 61.66107 |
| SOUTH KARACHI | 2021 | SINDH | 74.3971 | 60.59511 | 91.91049 |
| WEST KARACHI | 2021 | SINDH | 8.295522 | 4.446576 | 15.66282 |
| KASHMORE | 2021 | SINDH | 8.064516 | 4.08463 | 15.66474 |
| KASUR | 2021 | PUNJAB | 5.015045 | 2.172169 | 11.46244 |
| KECH | 2021 | BALOCHISTAN | 0 | 0.030084 | 3.431512 |
| KHAIRPUR | 2021 | SINDH | 7.687759 | 3.847263 | 14.97239 |
| KHANEWAL | 2021 | PUNJAB | 3.239741 | 1.19969 | 9.318378 |
| KHUSHAB | 2021 | PUNJAB | 9.411765 | 5.088278 | 17.15395 |
| KOHAT | 2021 | KPK | 0 | 0.022279 | 3.79458 |
| KORANGI KARACHI | 2021 | SINDH | 24.69087 | 16.6378 | 35.77642 |
| LAHORE | 2021 | PUNJAB | 65.26898 | 52.30472 | 81.81237 |
| LARKANA | 2021 | SINDH | 11.98692 | 6.930522 | 20.85521 |
| LASBELA | 2021 | BALOCHISTAN | 23.9521 | 16.30413 | 35.46762 |
| LEIAH | 2021 | PUNJAB | 9.594096 | 5.130782 | 17.97939 |
| LODHRAN | 2021 | PUNJAB | 0 | 0.025019 | 3.598253 |
| MALIR KARACHI | 2021 | SINDH | 17.96991 | 11.22132 | 27.70362 |
| MANDI BAHAUDDIN | 2021 | PUNJAB | 0 | 0.024175 | 3.334707 |
| MANSEHRA | 2021 | KPK | 4.618938 | 1.867643 | 10.99437 |
| MARDAN | 2021 | KPK | 0.725426 | 0.196062 | 5.276574 |
| MATIARI | 2021 | SINDH | 34.06326 | 25.04674 | 46.73427 |
| MIANWALI | 2021 | PUNJAB | 27.27273 | 19.43707 | 39.74183 |
| MIRPUR KHAS | 2021 | SINDH | 9.818731 | 5.305101 | 17.84056 |
| MULTAN | 2021 | PUNJAB | 1.023137 | 0.272424 | 5.811683 |
| MUZAFFARGARH | 2021 | PUNJAB | 0.024749 | 0.038093 | 3.933342 |
| NANKANA SAHIB | 2021 | PUNJAB | 0 | 0.023839 | 3.841903 |
| NAROWAL | 2021 | PUNJAB | 14.92537 | 9.342523 | 23.86775 |
| NAUSHAHRO FEROZE | 2021 | SINDH | 3.634476 | 1.360548 | 9.782569 |
| NOWSHERA | 2021 | KPK | 7.462687 | 3.487525 | 14.46241 |
| OKARA | 2021 | PUNJAB | 12.56281 | 7.248485 | 21.54991 |
| PAKPATTAN | 2021 | PUNJAB | 12.48699 | 7.667556 | 20.87657 |
| PESHAWAR | 2021 | KPK | 5.593087 | 2.521208 | 12.05628 |
| QUETTA | 2021 | BALOCHISTAN | 5.488474 | 2.421529 | 12.02163 |
| RAHIM YAR KHAN | 2021 | PUNJAB | 0.223105 | 0.04948 | 4.167635 |
| RAJANPUR | 2021 | PUNJAB | 0 | 0.023711 | 3.410035 |
| RAWALPINDI | 2021 | PUNJAB | 0.101841 | 0.045138 | 4.00128 |
| SAHIWAL | 2021 | PUNJAB | 0.517822 | 0.115935 | 4.479103 |
| SANGHAR | 2021 | SINDH | 2.972399 | 0.923769 | 8.55495 |
| SARGODHA | 2021 | PUNJAB | 0.514389 | 0.120809 | 4.49674 |
| SHAHEED BENAZIR ABAD | 2021 | SINDH | 0.679071 | 0.12712 | 5.156068 |
| SHEIKHUPURA | 2021 | PUNJAB | 0.489581 | 0.092223 | 4.499923 |
| SHIKARPUR | 2021 | SINDH | 6.23053 | 3.008755 | 13.09346 |
| SIALKOT | 2021 | PUNJAB | 0.193215 | 0.052252 | 4.238633 |
| SUJAWAL | 2021 | SINDH | 0 | 0.02777 | 3.284205 |
| SUKKUR | 2021 | SINDH | 18.34862 | 11.09035 | 28.44781 |
| SWABI | 2021 | KPK | 0 | 0.02084 | 3.268484 |
| SWAT | 2021 | KPK | 0 | 0.022496 | 3.613999 |
| TANDO ALLAHYAR | 2021 | SINDH | 2.531646 | 0.717968 | 8.263871 |
| TANDO MUHAMMAD KHAN | 2021 | SINDH | 0 | 0.020515 | 3.836943 |
| THARPARKAR | 2021 | SINDH | 285.7143 | 260.2988 | 312.8446 |
| THATTA | 2021 | SINDH | 8.695652 | 4.312936 | 16.51391 |
| TOBA TEK SINGH | 2021 | PUNJAB | 0.396079 | 0.091838 | 4.273602 |
| UMER KOT | 2021 | SINDH | 14.16431 | 8.822499 | 23.83481 |
| VEHARI | 2021 | PUNJAB | 2.44858 | 0.792963 | 7.819011 |
| ABBOTTABAD | 2022 | KPK | 0 | 0.021474 | 3.727597 |
| ATTOCK | 2022 | PUNJAB | 27.18447 | 19.14633 | 39.20932 |
| BADIN | 2022 | SINDH | 18.5567 | 11.94584 | 29.45669 |
| BAHAWALNAGAR | 2022 | PUNJAB | 0.276005 | 0.060722 | 3.868283 |
| BAHAWALPUR | 2022 | PUNJAB | 0.028717 | 0.018356 | 4.05596 |
| BANNU | 2022 | KPK | 0 | 0.031728 | 3.54767 |
| BHAKKAR | 2022 | PUNJAB | 4.201681 | 1.750408 | 10.17036 |
| CHAKWAL | 2022 | PUNJAB | 2.070393 | 0.72207 | 6.790234 |
| CHINIOT | 2022 | PUNJAB | 2.079002 | 0.657179 | 6.983717 |
| DADU | 2022 | SINDH | 18.73536 | 12.37438 | 29.65056 |
| DERA GHAZI KHAN | 2022 | PUNJAB | 6.10998 | 2.837486 | 13.04034 |
| D. I. KHAN | 2022 | KPK | 0.742942 | 0.167304 | 5.285559 |
| FAISALABAD | 2022 | PUNJAB | 0.897617 | 0.212702 | 5.409889 |
| GHOTKI | 2022 | SINDH | 52.5641 | 40.706 | 68.32351 |
| GUJRANWALA | 2022 | PUNJAB | 9.398844 | 4.897765 | 18.03972 |
| GUJRAT | 2022 | PUNJAB | 0.229323 | 0.072415 | 4.224721 |
| HAFIZABAD | 2022 | PUNJAB | 6.355932 | 2.902452 | 13.49594 |
| HYDERABAD | 2022 | SINDH | 99.48427 | 81.69437 | 118.2668 |
| JACOBABAD | 2022 | SINDH | 14.19355 | 8.5302 | 23.41096 |
| JAFFARABAD | 2022 | BALOCHISTAN | 14.92537 | 9.012281 | 24.09759 |
| JAMSHORO | 2022 | SINDH | 27.77778 | 18.73879 | 39.76956 |
| JHANG | 2022 | PUNJAB | 2.51004 | 0.856639 | 7.675869 |
| JHELUM | 2022 | PUNJAB | 1.017294 | 0.22334 | 5.57885 |
| KAMBAR SHAHDAD KOT | 2022 | SINDH | 46.83841 | 34.74519 | 61.56865 |
| CENTRAL KARACHI | 2022 | SINDH | 105.7148 | 87.17563 | 127.3877 |
| EAST KARACHI | 2022 | SINDH | 200.4132 | 176.4063 | 224.8696 |
| SOUTH KARACHI | 2022 | SINDH | 174.0214 | 151.1558 | 199.2256 |
| WEST KARACHI | 2022 | SINDH | 29.45708 | 20.84386 | 42.60294 |
| KASHMORE | 2022 | SINDH | 17.18213 | 10.7786 | 27.1716 |
| KASUR | 2022 | PUNJAB | 4.975124 | 2.330728 | 12.15249 |
| KECH | 2022 | BALOCHISTAN | 6.666667 | 3.507296 | 13.7745 |
| KHAIRPUR | 2022 | SINDH | 22.9682 | 15.18507 | 34.0326 |
| KHANEWAL | 2022 | PUNJAB | 9.120172 | 4.90465 | 17.13192 |
| KHUSHAB | 2022 | PUNJAB | 0 | 0.025868 | 3.962161 |
| KOHAT | 2022 | KPK | 1.222494 | 0.348807 | 6.295286 |
| KORANGI KARACHI | 2022 | SINDH | 63.47959 | 50.09838 | 79.6766 |
| LAHORE | 2022 | PUNJAB | 33.3264 | 23.81917 | 46.73105 |
| LARKANA | 2022 | SINDH | 23.07138 | 15.01117 | 34.44198 |
| LASBELA | 2022 | BALOCHISTAN | 313.6095 | 285.9537 | 342.6997 |
| LEIAH | 2022 | PUNJAB | 6.59824 | 3.356537 | 13.70957 |
| LODHRAN | 2022 | PUNJAB | 2.544529 | 0.905764 | 8.133763 |
| LORALAI | 2022 | BALOCHISTAN | 0 | 0.025227 | 3.613908 |
| LOWER DIR | 2022 | KPK | 0 | 0.029938 | 3.668302 |
| MALIR KARACHI | 2022 | SINDH | 85.3454 | 69.69552 | 104.3203 |
| MANDI BAHAUDDIN | 2022 | PUNJAB | 2.253944 | 0.763962 | 8.086272 |
| MANSEHRA | 2022 | KPK | 0 | 0.031319 | 3.735747 |
| MARDAN | 2022 | KPK | 3.962536 | 1.69271 | 9.982069 |
| MATIARI | 2022 | SINDH | 116.2228 | 97.87614 | 138.8347 |
| MIANWALI | 2022 | PUNJAB | 4.012036 | 1.638564 | 10.03342 |
| MIRPUR KHAS | 2022 | SINDH | 39.81968 | 28.98936 | 53.16389 |
| MULTAN | 2022 | PUNJAB | 7.166139 | 3.425886 | 14.49841 |
| MUZAFFARGARH | 2022 | PUNJAB | 0.294927 | 0.081226 | 4.323615 |
| NANKANA SAHIB | 2022 | PUNJAB | 0 | 0.014246 | 3.446178 |
| NAROWAL | 2022 | PUNJAB | 9.90099 | 5.618096 | 17.31615 |
| NAUSHAHRO FEROZE | 2022 | SINDH | 19.12145 | 12.65945 | 28.99183 |
| NOWSHERA | 2022 | KPK | 38.13559 | 27.84539 | 52.47565 |
| OKARA | 2022 | PUNJAB | 5.988024 | 2.89591 | 12.9031 |
| PAKPATTAN | 2022 | PUNJAB | 4.132231 | 1.91387 | 10.47136 |
| PESHAWAR | 2022 | KPK | 5.848868 | 2.515482 | 11.99622 |
| QUETTA | 2022 | BALOCHISTAN | 17.99346 | 11.57294 | 28.19105 |
| RAHIM YAR KHAN | 2022 | PUNJAB | 0.487437 | 0.092196 | 4.478007 |
| RAJANPUR | 2022 | PUNJAB | 0.987167 | 0.256422 | 5.287557 |
| RAWALPINDI | 2022 | PUNJAB | 1.052823 | 0.299383 | 5.581365 |
| SAHIWAL | 2022 | PUNJAB | 0.257224 | 0.056311 | 4.46943 |
| SANGHAR | 2022 | SINDH | 22.79443 | 15.37965 | 34.58626 |
| SARGODHA | 2022 | PUNJAB | 0.14182 | 0.035701 | 3.904795 |
| SHAHEED BENAZIR ABAD | 2022 | SINDH | 7.557355 | 3.646233 | 14.931 |
| SHEIKHUPURA | 2022 | PUNJAB | 0.698579 | 0.142892 | 5.069644 |
| SHIKARPUR | 2022 | SINDH | 9.287926 | 5.002202 | 17.66743 |
| SIALKOT | 2022 | PUNJAB | 0.356379 | 0.071084 | 4.49045 |
| SUJAWAL | 2022 | SINDH | 43.20988 | 32.22372 | 57.22744 |
| SUKKUR | 2022 | SINDH | 123.0647 | 104.9862 | 144.3704 |
| SWABI | 2022 | KPK | 22.72727 | 15.4922 | 33.68885 |
| TANDO ALLAHYAR | 2022 | SINDH | 18.89169 | 11.32159 | 29.54161 |
| TANDO MUHAMMAD KHAN | 2022 | SINDH | 16.21622 | 10.08633 | 26.23428 |
| THARPARKAR | 2022 | SINDH | 761.9048 | 734.6055 | 786.3263 |
| THATTA | 2022 | SINDH | 110.3896 | 92.31434 | 131.622 |
| TOBA TEK SINGH | 2022 | PUNJAB | 0.688637 | 0.151325 | 5.216256 |
| UMER KOT | 2022 | SINDH | 78.87324 | 64.08448 | 96.25526 |
| VEHARI | 2022 | PUNJAB | 2.915452 | 0.994372 | 8.076114 |
